# Impact of Routine Management Procedures on the Welfare of Suckling Piglets

**DOI:** 10.3390/vetsci9010032

**Published:** 2022-01-17

**Authors:** Simone M. Schmid, Julia Steinhoff-Wagner

**Affiliations:** Institute of Animal Science, University of Bonn, 53115 Bonn, Germany; simone.schmid@uni-bonn.de

**Keywords:** age impact, animal welfare, animal health, castration, marking for identification, pain management, piglet processing, processing technique, tail docking, teeth resection

## Abstract

Piglets often undergo several painful treatments during the initial days of their lives. In this review, we investigate the acute (i.e., immediate), short-, and long-term implications of piglet processing on behavioral, physiological, clinical, and performance parameters, and how welfare impairments depend on performance instead of sham procedure, alternative techniques, or the age of the piglets. Welfare indicators that have been used to determine the least distressing procedures and knowledge gaps with regard to the procedures are identified and discussed. Tail docking and especially piglet castration have been the most researched topics, whereas marking for identification has been rarely addressed. Few or no studies have investigated the effects of teeth resection and tail docking on piglets of different age groups. Additionally, results are often found to be inconsistent, highlighting the need for additional research to determine the optimal age for processing. Studies comparing different processing techniques have produced contradictory results, but ear notching, teeth clipping, hot cautery tail docking, and tearing during castration have been determined to result in increased pain. Generally, a shorter procedure duration can reduce stress, with operator training having a distinct impact on piglet welfare during processing. As such, these topics should be further investigated.

## 1. Introduction

Piglets have to undergo several treatments in the first days of life, collectively referred to as “piglet processing” [1,2,3]. Apart from the legally required marking for identification, piglets usually have to endure tail docking, teeth resection, and/or castration. Often, these procedures are performed in combination to reduce handling and work time [4,5]. However, all of the above-named procedures are considered painful [6,7], causing, at least to some extent and for some period of time, distress in newborn piglets. Acute stress, especially due to the performance of several routine procedures in a single session, can have negative effects on piglet welfare and on their subsequent production stages [5,7,8]. Conversely, if procedures are performed on different days, later procedures might induce a more intense pain reaction due to the previous pain experience [9].

In most cases, newborn piglets do not receive analgesia, nor anesthesia, to mitigate the suffering during these procedures [10,11,12]. To enable an assessment of changes in piglet behavior resulting from different management procedures, it is thus necessary to first describe the procedures in detail and elaborate on their potential implications on piglets’ health and welfare. Although, as has been previously claimed, millions of piglets have had to endure and adapt to the stress caused by piglet processing, alternative methods may improve piglet welfare, productivity, and acceptance [13]. It has been suggested that piglet processing procedures, if unavoidable, should be performed by an experienced stockperson to lessen the procedure’s duration, avoid errors, and minimize the stress the treatment might induce [13]. Several studies, which will be discussed throughout this review, have compared different procedures and techniques and assessed their impact on piglets of various ages in terms of pain and stress.

In this review, studies analyzing the acute response and long-term implications of marking, teeth resection, tail docking, and castration on piglet welfare are discussed. By doing so, the effects of the treatment itself, of alternative techniques, and of piglet age will be evaluated. Furthermore, it was aimed at providing an overview of applied welfare indicators. Additionally, knowledge gaps are highlighted, and open research questions are compiled for further studies in the future.

## 2. Materials and Methods

**Figure 1:**
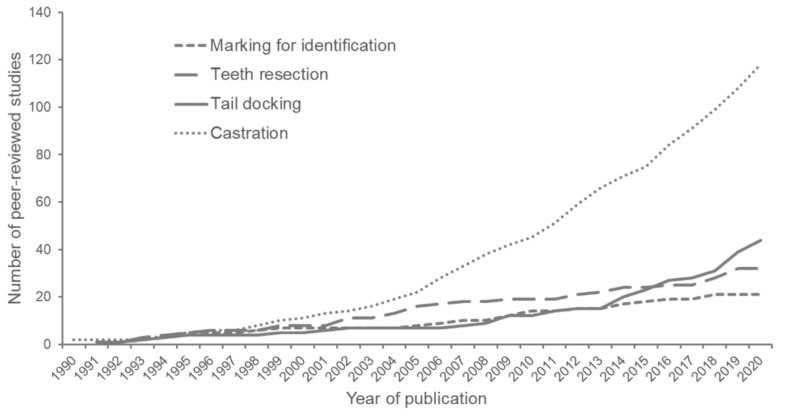
Cumulative number of peer-reviewed studies published between 1990 and 2020 that investigate the effects of identification (*n* = 21), teeth resection (*n* = 32), tail docking (*n* = 44), and castration (*n* = 118) on the welfare of suckling piglets. For the sake of completeness, this figure also shows studies that investigated the effects of anesthesia and/or analgesia and were not further discussed in the present review; however, processing studies performed in weaned piglets or adult animals were not included.

For this review, literature was searched on the databases pubmed.gov, webofscience.com and scholar.google.com. Key words for the literature search were ‘piglet’, the terms for the respective procedures (‘marking’ or ‘identification’ (i.e., ‘ear tagging’ or ‘ear notching’), ‘teeth resection’ (i.e., ‘teeth clipping’ or ‘teeth grinding’), ‘tail docking’, and ‘castration’), combined with other terms such as ‘pain’, ‘stress’, and/or ‘welfare’. Studies investigating the procedures in older pigs (e.g., weaned piglets or fattening pigs) were omitted. Only publications discussing the impact of the procedure itself, for example by comparing different treatment groups to sham handling groups, or by comparing different techniques or age groups, were included. In the case that only a few original research articles were found, other publication forms such as dissertations or conference papers that are available online were consulted. Only publications in English and German were included in the literature search. Studies published between 1990 and 2020 were researched, as is shown in Figure 1. For castration, 118 peer-reviewed publications were found, while the number for tail docking, teeth resection and marking for identification was lower (44, 32, and 21, respectively) (Figure 1).

**Figure 2:**
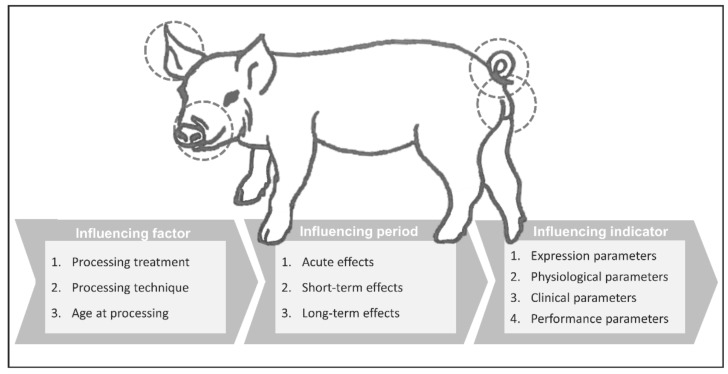
Influencing factors, periods, and indicators in the scope of the present review that describe the impact of piglet processing on animal welfare.

In the following chapters, each processing technique will be discussed individually. Each chapter contains three subchapters evaluating the impact of the procedure itself (by comparing its impact to sham handling groups), and further discussing the effect of different processing techniques and age groups. Each chapter closes with a summary of the found literature. Figure 2 shows the different influencing factors, periods, and welfare indicators that have been used in several studies to evaluate the impact of piglet processing on their welfare. As Figure 2 indicates, all publications were investigated not only for the above-named influencing factors of age and technique, but also for the time periods following the procedure and the applied welfare indicators.

## 3. Marking for Identification

Ear tagging has been identified as the most common among pig identification procedures; this is routinely performed in most European countries [2], whereas ear notching is often preferred in the USA and Australia [5,6,10,11,12]. Other animal identification options are tattooing or the implantation of transponders [13]. It has been suggested that, since there are several options for the identification of pigs, research should be focused on finding the method with the lowest potential for pain and distress [2,5]. An overview of the different identification options for pigs is presented in Table 1. The studies listed here did not focus on animal welfare issues of the different identification methods, therefore not all of them will be further discussed; however, they provide an overview of the possible marking options. Further, it has been determined that the readability of ear-notched identification marks can suffer due to incorrect execution or translation errors [14,15], but scientific studies on this welfare issue are lacking (Table 1). More details on the studies investigating the effect of marking for piglet identification have been provided in Supplementary Table S1.

### 3.1. Effects of Marking for Identification in Comparison to Sham Handling

Torrey et al. [3] compared the vocalizations and behavior of ear-notched and tail-docked piglets to sham-processed piglets and found more high-frequency calls in processed piglets when compared to sham ones. The authors concluded that processing is painful to newborn piglets [3]; however, since both identification and tail docking were performed, it is difficult to interpret the impact of each procedure. In a more recent study, the signal intensity and duration of vocalizations were higher in piglets marked by notching when compared to handled piglets [10]. Lomax et al. [11] have also noted a more intense behavioral response in notched piglets when compared to sham-processed ones. After ear notching, head shaking, and ear flapping have been predominately observed, which is explained by the irritation or pain caused by the cutting of the flesh from the ears [7]. In the immediate 10 min after ear notching (and tail docking), Torrey et al. [3] observed that processed piglets stood more and huddled less than the control piglets; however, this was also observed in sham-castrated piglets, which can be explained by the effects of handling and removing piglets from the pen.

Ear tagging has been shown to induce a low stress reaction with no changes to plasma lactate and ACTH, and an increase in plasma cortisol at 15 min post-tagging in gilts [25]. No comparable study with suckling piglets as subjects was found, however, it is possible that piglets exhibit a different stress response, which should be analyzed in further research. Stark [9] also observed an increase in the concentration of blood cortisol after ear tagging. Moreover, when comparing the responses of piglets being ear tagged and provided with and without analgesia, the non-analgesia piglets had more intense reactions, leading to the conclusion that ear tagging causes pain rather than only causing irritation due to a large object being attached to the ear. After ear notching, there was no effect of processing on piglet suckling behavior, mortality, or growth rates [3].

### 3.2. Effects of Alternative Identification Techniques

When comparing ear tagging to ear notching, the notching procedure was shown to take significantly longer to complete, which resulted in a longer handling duration in general [8,13]; this is one possible explanation for the increased squealing and higher peak frequencies that occur during notching [8,13]. However, these authors also found clear indications (e.g., changes in vocalization frequencies) for pain and stress during ear tagging [13]. Nonetheless, more escape attempts were observed in piglets undergoing ear notching when compared to those undergoing ear tagging. However, this was not replicated in a later study, in which ear-tagged piglets performed more escape attempts than control piglets, with notched piglets performing an intermediate number of escape attempts [8]. When ear tagging, ear notching, and the intraperitoneal injection of a transponder were compared, all three methods induced pain-related behaviors, as well as non-specific behaviors in piglets; however, pain-related activity was identified to be greater after ear notching and ear tagging [5].

When comparing the post-procedural effects of ear notching and ear tagging, a trend for higher cortisol values was noted in ear-notched piglets 4 h after the procedure [13]. After notching, more wounds and tissue damage were observed when compared to ear tagging [8]. Wound scores were also greater in the ear notching group 1 and 2 weeks post-procedure, indicating slower healing [13]. This was confirmed in a later study that revealed better lesion scores 24 (trend) and 48 h, as well as 1 week, after ear tagging when compared to ear notching [8]. After 2 weeks, the lesions of tagged piglets were only numerically better [8]. The authors concluded that notching impaired piglet welfare to a higher extent than ear tagging due to a longer duration of the procedure and increased tissue damage [13]. However, it remains unknown whether the tissue destruction caused by ear tagging also causes long-term distress [2].

At 21–28 days of age, Stärk et al. [21] tagged piglets with both a conventional and an electronic ear tag and injected them with one of two differently sized transponder systems (23 vs. 11.5 mm) at the base of their ears. Implantation of the smaller implant took more time, but resulted in the perforation of the skin in only few cases, whereas, in the case of the larger transponder, the removal of the injection needle resulted in an extraction of the chip in two cases [21]. Only 4% of these piglets showed signs of infection associated with transponder implantation. Wound healing was faster, whereas swelling and signs of infection were lower, when the larger transponder was implanted using an injection needle [21]. In a study by Bergqvist et al. [23], 1–2 and 9–10 week-old piglets were identified via tattoos, ear tags (conventional tags including electronic tags), and ear microchips (13 or 8 mm) injected into the auricle base. Lesions and ear tissue damage were evaluated after slaughter, which revealed significantly more wounds at the ear tag site than at the microchip injection site and that the pathological changes caused by the microchips were minimal [23]. Furthermore, the size of the microchip did not affect the incidence of wounds. However, chip size and piglet age affected the number of lost transponders, so the authors suggested that further research into the age at marking, chip type, and marking site is needed to improve this procedure [23].

### 3.3. Effect of Piglet Age at Identification

**Figure 3:**
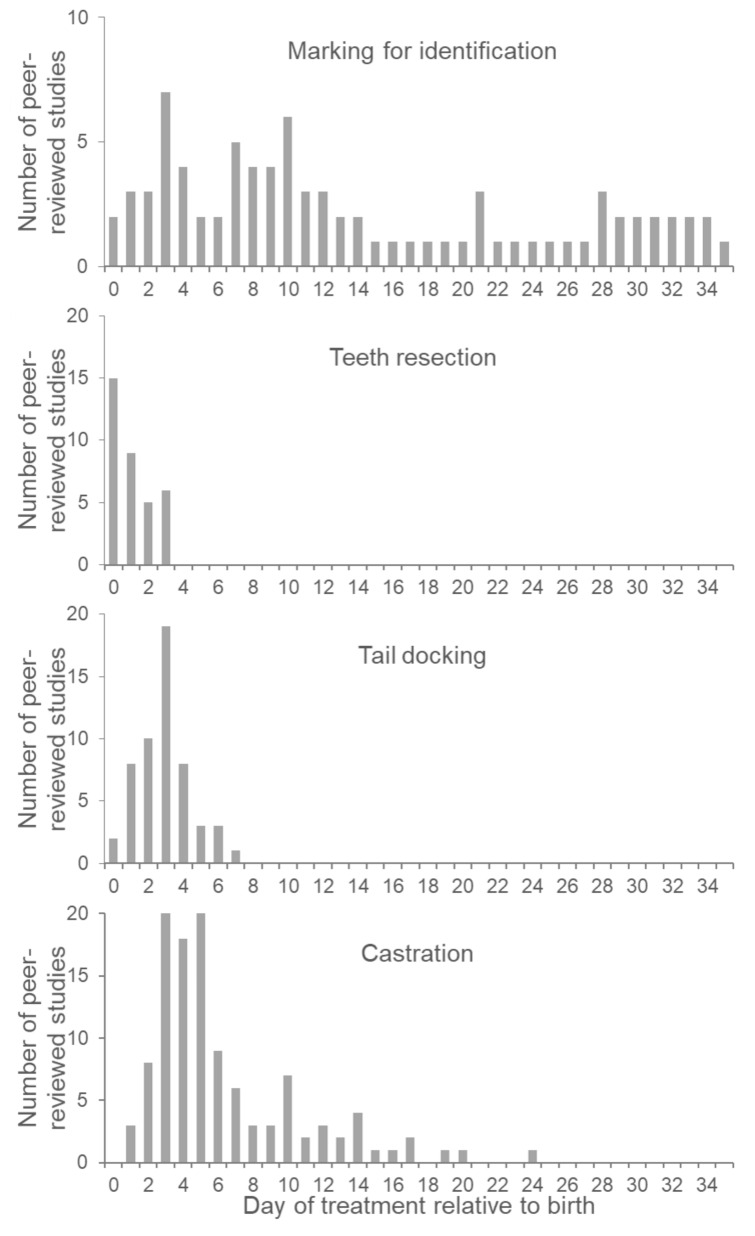
A frequency distribution of the timing of various processing treatments (marking for identification, teeth resection, tail docking, and castration) across the peer-reviewed studies included in this review.

Figure 3 illustrates the number of studies that performed each processing technique with respect to the piglet’s age. In contrast to other routine procedures, the marking of piglets for identification purposes is evenly distributed over the first few weeks of life. Several studies have also examined the marking procedures conducted among older pigs [18,23,26]. We assumed that the age of treatment selected in these field trials was mostly representative of the timing of the actual procedures on farms.

Regardless of their age (1 or 3 days), in the study by Torrey et al. [3], piglets that had undergone tail docking and ear notching were observed to have vocalized more when compared to sham-processed piglets; however, 1 day-old piglets produced significantly more high calls than 3 day-old piglets [3]. According to the authors, lying, standing, and tail posture were not influenced by piglet age at processing [3]; however, trembling was more pronounced in 1 day-old piglets and increased due to processing [3].

Torrey et al. [3] did not detect any effect of piglet age on IGF-I or IgA blood serum concentrations. However, concentrations of IgG, as measured at 5 days of age, were higher in piglets processed at 1 day old when compared to those processed at 3 days old, indicating a significant impact when piglets are processed on the third day after birth.

When performing ear notching, the depth of the notches should be monitored, as deep notches may enhance tearing of the ears [14]. Particularly when performing the ear notching in older and consequently larger pigs, notched pigs should be housed in separate pens to avoid attracting pen mates to the bloody notches, as this could cause ear biting [14]. However, with respect to ear tagging and microchip injections, piglet age (1–2 or 9–10 weeks) did not influence wound incidence or health in the trial by Bergqvist et al. [23]. Furthermore, the retention rate was higher in the older age group, possibly due to the larger-sized ears of the bigger animals at the time of injection [23].

Torrey et al. [3] did not find any differences in terms of suckling behavior between piglets processed at different ages (1 or 3 days), nor did they find any differences with regard to growth rates and mortality. Thus, ear notching (combined with tail docking) was not traumatic enough to affect growth rates, but only average-sized piglets were included in the study [3]. In a later study with low- and average-birth-weight piglets processed on day 1 or 3, mortality was higher in the low-birth-weight group, which led the authors to conclude that delaying processing until day 3 might be advantageous for low-body-weight piglets [27].

### 3.4. Summary of Identification Effects and Practical Implications

In most countries, pigs must be marked for food safety reasons; therefore, this procedure will likely never be omitted, regardless of the stress it might induce. Studies on the effect of identification on piglet welfare, especially on wound healing, growth, and vitality are lacking. While, for the indicator behavior, only studies focusing on ear notching have been conducted, results considering physiological parameters are only available from ear tagging studies. With regard to alternative identification techniques, ear tagging seems to be less disturbing than ear notching. The stress experienced after ear notching, as represented by the endocrine response and wound healing duration, appears to have a longer duration, but more data are needed to confirm this assumption. Identification transponders may be a good option, as few health issues have been reported to be associated with their use, but there is a lack of information on the appropriate age and injection size. Additionally, the best age at which to perform ear tagging and ear notching has yet to be determined, as the generated results remain unclear. Nonetheless, indicators such as the increased trembling of piglets on the first day after birth indicate that the stress of marking, in addition to the high amount of energy needed for life-preserving functions, might be extremely strenuous, thus reducing their viability. Since piglets must be marked prior to weaning and rehousing, the identification procedure could take place days or even weeks after birth to avoid any negative impacts; this has been confirmed in several studies that were quoted above and may be a viable strategy for reducing stress in newborn piglets and decreasing the incidence of non-readable markers due to low durability and injuries from torn-off ear tags.

## 4. Teeth Resection

It has been a common practice to resect piglet teeth [13,28,29]. This is usually done in the first days of life [2,30], often combined with other procedures, such as tail docking, during the course of piglet processing [31]. Teeth resection can be performed in two ways: clipping and grinding. Teeth clipping has been the method of choice for many years, but is becoming less popular [6,32] due to research findings and legal restrictions.

For teeth clipping, side-cutting pliers are used, and teeth are often clipped to the gum line [28,33]. This is critical, since the teeth substance, which can be removed without damaging the pulp chamber, is small (1.0–1.3 mm) [34]. Clipping teeth too close to the gum line can severely damage the teeth, providing an entrance pathway for pathogens and creating sharp edges, which can cause mouth wounds [29,35]. Often, an opening in the pulp cavity is observed after tooth resection [36]. Another approach is to only clip the tips of the teeth [33].

An alternative to clipping altogether is teeth grinding. For this approach, rotating electric grinders are used, which are said to minimize tooth damage [29,30]. Many studies have focused on the effects of teeth resection, including both immediate responses and long-term implications; these are outlined in Supplementary Table S2. Nonetheless, observations are often controversial, resulting in contradicting recommendations as to whether and how to resect piglet teeth [1,13,37,38].

### 4.1. Effects of Teeth Resection in Comparison to Sham Handling

Most studies investigating the effect of teeth clipping on piglet welfare have focused mainly on the time after the procedure. Some studies, however, have compared the immediate implications of the different resection methods. In general, piglets undergoing teeth clipping vocalize significantly more than sham-handled piglets [37]. After teeth resection, piglets often exhibit abnormal behaviors [39,40]. Piglets whose teeth have been clipped show one specific behavior, that is, teeth champing [7,29,39]. Teeth champing is assumed to be due to irritation from the teeth fragments and the presence of blood in the piglets’ mouths [7]. According to Lewis et al. [29], champing is not observed during the suckling period, indicating that this is an immediate and transient reaction to treatment. Furthermore, piglets with clipped needle teeth are found to be less active and tend to explore less on days 5 and 15 [41]; in this particular study, all piglets also had their ears notched and their tails docked, which might have increased the pain the piglets were experiencing. Furthermore, individual and social play behavior was also observed to lessen after teeth resection on days 5 and 15 [41]; similar findings were later published by Zhou et al. [37] and Fu et al. [38].

Teeth grinding has been determined to decrease the number of piglet face injuries [31]. Additionally, Lewis et al. [29] and Bates et al. [42] observed a decrease in face injuries in both ground and clipped piglets when compared to piglets with intact teeth. In an outdoor production system, the same effect was found after tooth clipping [43]. However, in a more recent study, face lesions as a result of intact teeth occurred in only one litter, supporting the call for non-routine teeth resection [1]. Additionally, even if teeth resection can reduce face lesions, lip lesions may also increase [29,39]. An additional issue is the risk of open pulp cavities due to teeth resection. Hessling-Zeinen [34] found that, after grinding, about 90% of examined teeth had at least one open pulp chamber, which not only causes immediate pain, but may also result in excruciating long-term pain due to inflammation.

In 30 studies (see Supplementary Table S2), the impact of teeth resection on the well-being and performance of piglets in the days and weeks following the procedure has been evaluated. As reviewed by Marchant-Forde et al. [13], the effects on production seem to be diverse; one reason for this may be the effect of the different operators themselves, which may have an influence on study results [31]. When performing selective tooth clipping in larger piglets only, the weight gain of low-birth-weight, unclipped piglets increased only slightly in medium-sized litters, or not at all in large-sized litters [44]. Particularly in low-birth-weight piglets, but also in others, teeth resection failed to increase weight gain [37,41,45,46]. On the contrary, weight gain in the first days of life may even be reduced by teeth clipping [33] or grinding [13,30]. In the weeks following treatment, weight gain has been observed to decrease in clipped piglets, which can be explained by possible fractures, bacterial colonization, and tooth pain, hindering milk intake [1]. In a study by Menegatti et al. [1], piglets with ground teeth showed the largest weight gain in the first days after treatment, possibly due to the absence of lesions and more relaxed sows. In a study by Gallois et al. [46], there was no effect of treatment on growth rates. Moreover, Brown et al. [43] did not detect any effect of the treatment on health status; this has been confirmed by other authors as well [35]. Bates et al. [42] noted higher nursing mortality rates in clipped piglets than in intact piglets, whereas Hansson and Lundeheim [47] observed an opposite effect, noting fewer mortalities in ground piglets when compared to intact piglets. Menegatti et al. [1] noted that multiple factors can influence health indicators such as mortality rate. Nonetheless, several authors have noted no effect of teeth resection on mortality rates [1,29,31,37,41,42,48].

### 4.2. Effects of Alternative Teeth Resection Techniques

Sinclair et al. [49] did not detect any consistent treatment effects of teeth clipping and grinding, both in the short and long-term, on piglet behavior. In a later study, however, Sinclair et al. [40] observed significantly more champing in clipped piglets, but also noted champing in ground and handled piglets, similar to the observations made by Meunier-Salaün et al. [39]. After both clipping and grinding, piglets walk and explore less, their ear position changes, and head flicking increases; however, indicators of pain are more obvious in clipped piglets [40]. Additionally, grinding takes significantly longer to complete than clipping, which increases the handling duration and, thus, increases the stress imposed on piglets [13,28,29,32,50,51]. Conversely, Ellert [32] noted that grinding using both a conventional grinder and a newly developed teacup grinder was much shorter in duration. As such, the stockperson performing the procedure and the equipment involved can either have a positive or negative impact [31,32], such that well-trained persons may be able to increase the speed and efficiency with which the treatment is carried out.

In terms of behavior, Marchant-Forde et al. [13] observed more escape attempts in piglets that have undergone grinding and further noted that piglets who had experienced grinding had higher frequency and longer call durations, indicating a more intense reaction to the procedure; hence, the grinding procedure impaired well-being to a higher extent than clipping [13]. Similar results were also obtained in a later study [8]. On the other hand, Llamas Moya et al. [28] claimed that grinding elicited a lower amount of acute phase proteins on day 29 than clipping. Meanwhile, Lewis et al. [29] did not find differences in the suckling or agonistic behavior of clipped, ground, or intact piglets; however, ground piglets were found to stay close to the sow’s udder immediately after the procedures, suggesting that these piglets may have had a more stressful experience due to the noise, heat, and duration of grinding; this would also make ground piglets more prone to crushing by the sow [29]. Conversely, on the days following the procedure, clipped piglets used the nest less and generally slept longer, which could be linked to possible infections in this group [29].

Furthermore, both clipped and ground piglets experienced a decrease in their skin temperature immediately after the procedures, which was caused by stress due to the teeth resection, leading to a reduction in blood flow to the skin [28]. The authors assumed that both pain due to the actual procedure and stress due to handling and restraint played a significant role in such an observation [28]. Although they categorized teeth resection as a transient stressor, since skin temperatures normalized 10 min after the procedures, they nonetheless recommended the provision of extra heat sources in farrowing crates to mitigate the effect [28]. Fu et al. [38] also observed a reduction in body surface temperature following clipping.

With respect to cortisol levels, results were found to differ, with some studies showing higher cortisol levels in piglets undergoing grinding [13,28], while others noting no effect after clipping or grinding [52]. Furthermore, Llamas Moya et al. [28] compared the effects of teeth clipping and grinding on the concentrations of C-reactive protein, serum amyloid A, and cortisol in piglets, concluding that both caused comparable inflammatory responses. In clipped piglets, teeth fractures may lead to elevated C-reactive protein levels, which are considered a non-specific marker of inflammation [28]. Llamas Moya et al. [28], however, did not observe any acute phase response to teeth resection 24 h after treatment. Piglets undergoing teeth resection via grinding were determined to have higher β-endorphin concentrations 4 h after treatment, and, 1 week later, they tended to have elevated cortisol values when compared to clipped piglets [13]. Sinclair et al. [53] found evidence of inflammatory tooth pain, namely, increased mRNA expression of the pro-inflammatory cytokine CXCL8, in piglets up to 6 weeks after the resection procedure; they concluded that clipping, and to a lesser extent grinding, impairs tooth integrity. On the other hand, Menegatti et al. [1] assessed serum protein 10 days after the procedures and did not find any effect of clipping or grinding, indicating that there were no infections in either group.

A disadvantage of grinding is the potential development of heat during the procedure, which can cause pain and possibly result in pulpitis [50]; however, the risk of injury and the occurrence of teeth fractures or bleeding seem to be higher with teeth clipping [35,36,46,50]. Often, more tooth substance is removed during clipping than grinding [50]. Splintered, clipped teeth can result from blunt clippers or poor technical abilities [35], with splinters linked to gingivitis and lip lesions in clipped piglets’ mouths [50], but infections can likely go unnoticed [35]. In one study, pulpitis was observed in half of the ground teeth, whereas signs of pulpitis were observed in almost all clipped teeth [50]. Apparently, pulpitis can occur even when the pulp chamber is not opened, depending on the damage to the tooth substance, the instruments used, and the duration of the procedure [32]. In a study conducted by Sinclair et al. [53], pulp exposure was detected in the teeth of all clipped and ground piglets, with minor bleeding occurring in a few piglets in the grinding group, but in almost all clipped piglets [40].

Later, during the suckling period, facial lesion scores have been found to be lower in clipped piglets than in ground ones [29,35]. The reduction of skin lesions noted in these studies, as well as by Weary and Fraser [33], was not linked to a higher weight gain; however, Hutter et al. [50] noted an increase in daily weight gain during the first week of life and lower mortality in piglets that had experienced teeth grinding when compared to those that had undergone clipping. In another study, grinding also tended to have a negative influence on piglet growth rate and weight 2 weeks after the procedure [13]. According to Holyoake et al. [30], the average weaning weight of clipped piglets was higher than that of ground piglets, and there were fewer preweaning deaths recorded in piglets in the clipped group. This result was, however, not confirmed by Lewis et al. [29] or Gallois et al. [46], who found no effect of teeth resection on weight development.

### 4.3. Effect of Piglet Age at Teeth Resection

Kober and Thacker [54] found that mortality was lower and piglet weaning weight was higher in piglets processed on day one when compared to those processed on day two; however, these authors have noted that the optimum processing day may differ between farms. To the best of our knowledge, no additional studies have investigated the effect of teeth resection on different age groups. In most of the studies included in this review, teeth resection was performed on the day of or within 24 h of birth, but not later than the third day of life (Figure 3). When compared to other processing treatments, teeth resection is usually applied first. This highlights the prophylactic aspect of this procedure. It would be preferable to wait for a necessary intervention and support natural tooth attrition by, for example, supplying piglets with manipulable toys.

### 4.4. Summary of Teeth Resection Effects and Practical Implications

Several studies have investigated the impact of teeth resection, both generally and in terms of the techniques of grinding and clipping. Regardless of the technique, teeth resection has been determined to result in increased vocalizations and altered piglet behavior, indicating a reduction in piglet well-being. Teeth champing is one behavior that appears to be specific to teeth resection. Several studies claim that altered behavior as a result of teeth resection, as well as physical reactions such as drops in temperature, are short-lived, indicating a relatively low impact of the procedure; however, other studies have provided contradictory results.

After reviewing all studies, it remains unclear whether teeth resection reduces injuries. Face lesions have been observed to decline in most cases, but lip lesions can increase, mostly in piglets undergoing clipping, and often results in inflammatory responses that are noted even weeks after the procedures. Generally, clipped piglets demonstrate indicators of pain and many cases of open pulp chambers, gingivitis, and pulpitis. Piglets that underwent grinding also had open pulp chambers.

The duration of the procedure itself appears to depend on the training of the operator; in some cases, grinding was reported to take longer, while in other trials, clipping took longer to complete. Nonetheless, a longer procedure duration can result in more distress. Also, the effects of resection on production parameters have remained inconsistent, probably as a result of the diversity of trial settings and assessment procedures. Some authors have reported no effect of teeth resection on weight gain or mortality. The effect of piglet age at processing has not been investigated, suggesting that there is no need to undertake teeth resection at an older age, since it is performed either in the first days or life or not at all. In the end, since it is forbidden in Europe to resect teeth prophylactically, research on other preventive measures is highly encouraged.

## 5. Tail Docking

Tail docking is performed routinely worldwide to prevent tail biting [13,55,56,57,58,59]. In the EU, over 90% of pigs are tail-docked, despite a ban on routine docking [60,61]. Tail docking means that a part of the tail is cut off, usually without pain mitigation [59,62]. It is usually performed in the course of piglet processing in the first days of life, often together with other procedures, such as teeth resection [31].

Several tools are used for tail docking, such as teeth clippers, pliers, scissors, scalpels, or cautery irons [59]. An additional method involves rubber ring tail docking, during which a constrictive rubber ring is attached to the tail [63]. Side clippers were traditionally used for tail docking, but, nowadays, heated clippers have become more popular as they cauterize and seal the wound, reducing the risk of infection [13]. As shown in Supplementary Table S3, several studies have compared the immediate behavioral and physiological reactions of piglets undergoing tail docking, compared to non-docked control groups or pigs docked using different techniques, and have found that tail docking is painful for piglets [2,7,13,37,63,64].

### 5.1. Effects of Tail Docking in Comparison to Sham Handling

Piglets that have undergone tail docking (and other procedures) have been shown to be in distress and in acute short-term pain, as indicated by changes to their vocalizations and behavior when compared to handled control piglets [3,37,65]. Noonan et al. [7] observed more grunting during and immediately after tail docking when compared to control piglets. There is no difference between male and female piglets with regard to the effect of tail docking [65]. Tail-docked piglets were observed to vocalize more, louder, and at a higher frequency and intensity [3,66]. Marchant-Forde et al. [13] also compared vocalizations and found that tail-docked piglets (regardless of the technique used) vocalized with higher peak frequencies than undocked control piglets. When comparing the vocalizations of piglets before and during tail docking, the percentage of stress vocalizations was higher during the procedure [58].

With respect to body movements during the procedure, Tallet et al. [66] noted more twisting and leg movements and less relaxation in docked piglets, which was interpreted as an expression of stress and pain. Tail jamming is one abnormal behavior found in tail-docked pigs, which is likely indicative of stress [7]. Additionally, tail wagging is more frequent in tail-docked pigs and is therefore characterized as an indicator of pain in the tail region [7]. Tail jamming was also observed by Torrey et al. [3]; however, although tail jamming was numerically higher on the first day after the procedure, it did not significantly increase until the third day, with no treatment effect on tail wagging [3]. A more recent study also noted lower and more tucked tails immediately after the procedure, although this was not a statistically significant difference [67]. This was confirmed by Tallet et al. [66], who found increased tail immobility in the first hours post-procedure and additionally in the following weeks until weaning, but with the tail held horizontally rather than in a low posture, which was interpreted as a response to inflammation and pain [66]. Environmental temperature might have contributed to this behavior, as more hanging and tucked down tail positions were noted at lower nest temperatures [68]. An additional abnormal behavior was observed in a study by Sutherland et al. [63], that is, tail-docked piglets (regardless of the technique) spent more time scooting than intact piglets, for up to 45 min after the procedure. Thus, the authors suggested that scooting has helped these piglets to reduce the discomfort caused by the procedure since this behavior was not observed before tail docking. Altogether, results suggest that tail wagging and jamming, scooting, and vocalization are suitable behavioral indicators of tail docking stress in piglets, with the occurrence of sitting after the procedure being unconfirmed as evidence [63]. However, tail wagging may be excluded, since its suitability as an indicator for pain remains unclear, as summarized by Yun et al. [69] and Vitali et al. [68].

Herskin et al. [64] have also claimed that tail docking may have a long-lasting effect on piglets, as they noted behavioral changes during a 5 h post-procedure observation period, with increases in behavioral alterations during the fifth hour. Docking length has been determined to have an effect on trembling, tail flicking, and scooting (trend), with no clear influence pattern identified [64]. An additional behavior indicative of distress is isolated lying, which was higher in pigs that were tail-docked without anesthesia when compared to intact pigs and pigs that received pain relievers [37,38,58]. Differences in lying were not detected from 5 to 15 days of age, suggesting only temporary distress immediately following the procedure [37]. Additionally, Tallet et al. [66] observed more lying in the first hours after tail docking, as well as from day six until weaning, which was interpreted as a possible protective reaction. By contrast, Torrey et al. [3] observed less lying and more standing in processed piglets immediately after the procedure, with no difference in the time spent lying alone or sitting. Di Martino et al. [70] have also found increased lying and inactivity, as well as decreased exploratory behavior, in undocked pigs during the rearing and fattening phase. In addition to the above-named behaviors, Tallet et al. [66] also noticed changes in ear posture and increased ear movements after tail docking, which were considered indicators of pain. In a novel approach, the facial expressions of tail-docked and undocked piglets were analyzed, and a change in “orbital tightening” was identified as a possible indicator for post-procedure pain [67]. The piglet grimace score was also applied by Viscardi and Turner [65], who observed significantly more grimacing in piglets that were tail-docked without pain relievers when compared to piglets that received pain treatment or were handled only. Tail docking has been noted to have no effect on tear staining, and it was assumed that the distress caused by tail docking may be too short or too low to have an impact [66]. This was, however, opposed by Vitali et al. [68], as they noted a higher tear staining score in tail-docked piglets. They explained the different study results, inter alia, by differences in the experimental designs.

There were no differences in cortisol and β-endorphin levels between docked and undocked piglets at several time points, from 45 min until 2 weeks post-procedure [13]. After 90 min, no differences were observed in terms of cortisol concentrations between the treatment groups, and since piglet behavior did not differ, the authors concluded that the stress experienced by tail docking was transient [63]. These results were confirmed in a later study, which revealed higher cortisol values in tail-docked pigs 30 min after the procedure, but similar concentrations 60 and 120 min post-procedure [58].

Based on several blood samples taken within the first 2 h after tail docking, Sutherland et al. [58] found that lymphocyte counts were reduced in all tail-docked pigs, regardless of anesthesia treatment, when compared to handled piglets. Furthermore, the neutrophil-to-lymphocyte ratio was higher in docked piglets (except for piglets docked under CO_2_ anesthesia) than in handled piglets [58]. As an explanation, the authors suggested leukocyte trafficking, in which lymphocytes are reallocated to organs or lymph nodes as a response to pain and stress induced by the docking procedure [58]. However, there were no differences in terms of acute phase response, as measured by C-reactive protein, and the total white blood cell count between the different docking groups at the time of weaning, indicating that there was no inflammation (i.e., no infection) a few weeks after the procedures [71]. Di Martino et al. [70] collected several blood samples from docked and undocked pigs during the weaning and fattening phase to measure haptoglobin and cortisol concentration, as well as the albumin/globulin ratio. Although tail lesions were increased in the undocked group, they found no significant differences between the two groups, which led to the conclusion that no subclinical inflammation was present [70].

Sandercock et al. [72] did not find a long-term effect of tail docking on nociception 4 to 5 weeks after the procedure and therefore assumed that its impact is restricted to the duration required for healing. Di Giminiani et al. [73] observed long-term hypersensitivity in tail-docked pigs and concluded that tail amputation can induce sustained changes in the peripheral mechanical sensitivity. Recently, tail amputation was shown to cause sustained changes to the expression of approximately 3000 genes up to 16 weeks after the procedure [57]. Several of these genes were determined to be involved in the inflammatory and neuropathic pain pathways, leading to the assumption that tail docking may have long-term implications for pig health and welfare (e.g., hypersensitivity in the distal stump) and could go unnoticed later in life, since pain-related behavior is most closely followed immediately after the actual procedure. However, Tallet et al. [66] noted increased reactions due to oral contact in docked piglets until weaning. As a positive side effect, Simonsen et al. [74] and Sutherland and Tucker [59] suggested that these more sensitive piglets may fight off biting attempts at an early stage, implying that only a part of the tail needs to be removed [71].

In the first 24 h after tail docking, the body weight of docked and intact piglets did not differ [58]. Additionally, Torrey et al. [3] did not find any effect of tail docking on suckling behavior or growth, which was further confirmed by Tallet et al. [66] and Fu et al. [38]. In another study, the mean bodyweight of processed pigs was determined to be lighter than that of handled pigs at 21 days of age; however, no differences were noted at 70 and 160 days [37]. Moreover, backfat depth and lean muscle yield were similar [37]. Sutherland et al. [71] found conflicting results: no differences in body weight and daily gain were found at weaning, but at 7 weeks of age, tail-docked pigs weighed more than the intact pigs. At this time, the tail biting lesion scores were also higher in intact pigs and pigs with longer docked tails, indicating reduced welfare and potentially explaining the lower body weight gain [71]. There was no difference in mortality between docked and undocked animals [38,70]. According to Simonsen [75], nibbling, tail biting, and the relative growth rate during the fattening period were not affected by tail status (docked or intact).

### 5.2. Effects of Alternative Tail Docking Techniques

Several experiments conducted by Marchant-Forde et al. [8,13] showed that tail docking with gas-heated cautery clippers took longer than docking using side-cutting pliers, which resulted in more stress to the piglet due to the longer duration of handling. The authors noted more escape attempts during hot docking, emphasizing that performance needs to be more precise to avoid accidental burning [8]. Furthermore, piglets tail-docked with hot cautery clippers vocalized significantly longer, including more squealing and at a higher frequency, than piglets docked with cold pliers, but with both groups vocalizing more than controls [13]. The authors concluded that using hot cautery clippers for tail docking results in a significant impairment of piglet welfare, which can be attributed to the longer handling duration and the possible additional pain due to accidental contact of the hot iron with the tail before the docking, causing burns; this happened frequently as the piglets tried to escape the restriction [13]. In a later study, both hot and cold tail-docked piglets vocalized at a higher frequency than sham-handled piglets; however, these piglets were all subject to additional procedures, such as identification, teeth resection, and castration [8]. Additionally, docking length appears to have an effect on the pain response of piglets, as increased squealing is observed when removing more tail tissue [64].

Sutherland et al. [63] compared tail docking via conventional blunt trauma cutting using cutting pliers and by heated cautery irons. They found higher cortisol concentrations 60 min after the procedures in piglets that were conventionally tail-docked (blunt trauma cutting) when compared to piglets tail-docked with a heated cautery iron; the cortisol values of the latter group were similar to those of undocked piglets. Thus, the authors assumed that using a heated iron resulted in burns destroying the nociceptors, which, in turn, led to reduced pain perception; however, they were uncertain whether these piglets experience more distress than conventionally tail-docked piglets once the nociceptors have regenerated [63]. This result was not confirmed by Lecchi et al. [76], who noted higher salivary cortisol concentrations in piglets that had been tail-docked with a gas-heated iron when compared to sham piglets 30–45 min after the procedure, but these piglets had also been castrated. In another study, no differences were observed in terms of cortisol and β-endorphin levels between hot and cold docked piglets at several time points from 45 min until 2 weeks post-procedure [13].

There were no differences between blunt cutting and docking with a cautery iron with respect to wound healing [13,63], but in later publications, these same authors found that the wounds of pigs that had been tail-docked with cautery irons healed slightly slower [71] and thus had worse lesion scores [8]. In a study comparing different anesthesia treatments and docking with pliers, there were no differences in wound healing scores across all piglets [58]. In the stumps of tail-docked fattening pigs, neuromas were detected at the time of slaughter, with docking having taken place with an emasculator [74], as well as with a cautery iron at different docking lengths [77]; it has been assumed that these pigs had increased sensitivity to pain at the tail and suffered from chronic discomfort and long-term pain [59,74,77]. In piglets tail-docked with a cautery iron, growth was observed to have reduced in the second week post-procedure [13]; this was not the case for piglets that had been blunt tail-docked nor undocked control piglets. Nonetheless, the authors were uncertain whether the docking technique had a real effect on weight development since other parameters were unaffected.

### 5.3. Effect of Piglet Age at Tail Docking

As is evident from Figure 3 and Supplementary Table S3, tail docking is usually performed during the first week of life, with a significant proportion of published studies conducting tail docking on the third day after birth. As it is assumed that trial designs reflect practical conditions, this indicates that, similar to teeth resection, tail docking is performed prophylactically rather than due to the incidence of actual biting, as the onset of tail biting usually occurs weeks later [60]. The only studies that have examined the potential effect of age on tail docking are those conducted by Torrey et al. [3], Bovey et al. [27], and Sandercock et al. [57]. They compared the impact of tail docking among 1 and 3 day-old piglets and found that vocalizations were of a higher frequency in 1 day-old docked piglets when compared to 3 day-old docked piglets [3]. Behaviors such as lying and standing were not influenced by age at docking, but more trembling was observed in the younger group. Processed low-birth-weight piglets spent more time dog-sitting and less time lying than heavier piglets, and playing was more observed to be frequent in piglets processed on day three [27]. Suckling behavior remained unaffected. Torrey et al. [3] did not detect any effect of age at tail docking on IgG serum concentrations, which were noted to decrease in all treated piglets when compared to control piglets. Moreover, body weight at 5 and 14 days of age was not influenced by the age at processing, nor treatment in general [3]. The authors concluded that neither processing on day one nor on day three is advantageous. When including low-birth-weight piglets in a subsequent study, body weight at weaning was still lower in low-birth-weight piglets, but there was no effect of processing timing (day one or three; [27]. The age span that was investigated in the study by Sandercock et al. [57] was larger, with piglets being 3 and 63 days old; however, in both age groups, there were several changes in gene expression when compared to sham-handled piglets.

### 5.4. Summary of Tail Docking Effects and Practical Implications

As Tallet et al. [66] have claimed, a number of studies have focused on the welfare consequences of leaving pig tails intact, with less attention being paid to the long-term consequences of the tail docking procedure. Nonetheless, 36 studies (see Supplementary Table S3) have produced results of the impact of tail docking. Altogether, results suggest that vocalizations, tail jamming, and scooting are suitable behavioral indicators of tail docking stress among piglets. Additional recent approaches include the piglet grimace scale and tear staining score, with claims that grimacing (i.e., pain) increases due to tail docking. Most authors agree that behavioral changes are temporary, indicating that the effect of tail docking is rather short-lived; this has been confirmed by the immediate, but short-lasting, increases in cortisol levels among docked piglets and the lack of inflammatory signs later in life. Nonetheless, there is evidence of long-lasting hypersensitivity after tail docking, which indicates chronic pain in processed piglets. With regard to growth and mortality, however, there are a few differences between undocked and docked piglets, indicating reduced weight due to biting incidents in undocked piglets. It is assumed that these observations highly depend on the study designs and farm management practices.

Considering the different docking techniques, results are not completely clear. Overall, the hot cautery method seems to impair welfare to a higher extent due to its longer duration, the potential for accidental burns, and slower wound healing, which again highlights the importance of having a well-trained operator. However, cortisol responses after applying cautery were not consistent across different studies. Furthermore, pain seems to increase with the length of the amputated tail. Only one study addressed a potential age effect, but found no clear impact in terms of behavior and growth after tail docking; since the investigated age span was small, subsequent studies with an age gap of 5 days or more could reveal differences. However, it needs to be considered that performing tail docking prophylactically during the first days of life is illegal in many countries.

## 6. Castration

According to the Panel on Animal Health and Welfare, approximately 100 million male piglets (80%) are castrated each year in EU member states [78]. In the USA alone, almost 100% of male pigs kept for food production are castrated [79]. Female pigs are also castrated, albeit less frequently, but little is known about its extent and applied techniques [78]. Thus, this review will focus on the castration of male piglets.

Two techniques are frequently applied for the severing of the spermatic cords: pulling of the cords until they tear off or cutting of the cords using a scalpel [4,80,81]. Other methods include twisting of the spermatic cords, cutting with scissors, or disconnecting the cords using an emasculator [4,81,82]. In general, there are several different recommendations for the castration, its preparation and post-surgical treatment, which can only result in different practices across farms, thus providing opportunity for optimization [83]. Particularly in more recent years, several studies have been conducted to describe the effects of castration on animal welfare, as can be seen in Supplementary Table S4 and Figure 1. Many authors have found that castration is a painful procedure [82,84,85,86,87].

### 6.1. Effects of Castration in Comparison to Sham Handling

Many studies have compared different treatment groups, such as handled piglets, castrated piglets, and piglets treated with some form of pain treatment before castration, to evaluate the effects of castration in general and the efficiency of analgesics or anesthetics. In the following section, we will focus on studies comparing sham-castrated and castrated piglets without pain treatment.

According to the European Food Safety Authority, short EFSA [78], “physiological and behavioral reactions indicative of pain are numerous during the process” of castration. During castration, Weary et al. [85] found that vocalizations were most intense during the later stages of the procedure, indicating that severing the spermatic cords is the most painful, whereas calls from sham piglets declined over time. This was confirmed by Taylor and Weary [86] and Marx et al. [88]. Additionally, Taylor et al. [89] observed an effect of castration on high-frequency, low-frequency, and total call rate, but none on the duration or frequency of calls. In accordance with these results, Marx et al. [88] observed significantly higher screams in the castration group, in addition to other parameters, such as longer duration or higher call energy, being affected. They interpreted an increase in screams to be indicative of an increase in stress, but opposed the thoughts of White et al. [84], who suggested that an increase in frequency was a sign of increased stress. They have also suggested that different restraining methods may have produced these varied results [88]. Additionally, Puppe et al. [90], Schön et al. [91], and Leidig et al. [92] identified that welfare was impaired during castration, due to the longer and higher frequency vocalizations in castrated piglets. According to their results, castration clearly evoked acute pain, resulting in complex alterations of the recorded vocalizations when compared to pre- and post-surgical periods. They have further assumed that piglets perceive castration as a highly threatening situation, comparable to a predator attack, to which they react with maximal vocalizations. Sutherland et al. [93], Kluivers-Poodt et al. [94], Sutherland et al. [87], Marchant-Forde et al. [13], Marchant-Forde et al. [8], and Sutherland et al. [95] confirmed this reaction, as they also observed more stress vocalizations in castrated than in sham piglets.

Apart from vocalizations, behavior changes can also serve as indicators of pain experienced during castration. Keita et al. [96] noted front and hind leg movements, as well as trembling, defecation, and urination. Furthermore, more escape attempts were observed in piglets undergoing castration [13]. Sutherland et al. [95] also noted increased limb and back movement in castrated piglets. Some scientists have suggested using facial expressions (e.g., cheek and orbital tightening) for the assessment of pain during and after castration [97,98,99]; however, the piglet grimace scale does not always reveal differences between castrated and sham-castrated piglets [98], with only later castrated piglets (with and without analgesia) grimacing significantly more than the sham-castrated ones [99]. An additional altered behavior was reported after castration, that is, the piglet sliding themselves across the floor [100]. All these studies have similarly shown that abnormal postures and behaviors usually disappear within a few hours of the procedure, so it is assumed that any changes after the acute phase are different and not easily detectable [80]. When extending the observation time, Hay et al. [80] found reduced suckling behavior and udder massages, but more walking in castrated piglets during the first hours after castration. A tendency for this behavior pattern was also found by Stark [9], who argued that castrated piglets may be distracted by the pain, whereas other studies found no differences in the first hours after the procedure [89,101]. Behavior changes were noted in the following hours, with more castrated piglets lying at or massaging the sow’s udder [89,102]. On the first day after the procedure, castrated piglets were increasingly isolated, desynchronized, and inactive while awake, demonstrating pain-like behaviors, such as stiffness, exhaustion, and trembling [80].

Kluivers-Poodt et al. [103] have also detected more pain-related behavior in castrated piglets on day one, but not on the following days. In contrast, activity, scratching, and tail wagging were observed to increase on the second day in the studies conducted by Hay et al. [80] and Llamas Moya et al. [102], with scratching continuing over the four following nights. Kluivers-Poodt et al. [103] found no difference in tail wagging behavior. Throughout the whole trial, castrated piglets kneeled more, a behavior that had not been detected in earlier studies [80]. Thus, some behaviors are limited to the acute phase after castration, while others are more pronounced in the following days, indicating a longer pain experience [80]. Differences between study results were also explained by the performance of other events, such as tail docking, shortly after birth in the studies by Hay et al. [80] and Llamas Moya et al. [102], which may have caused an altered pain response and an increase in stress [103].

Another indicator is tail movement, which is observed to increase during or after castration [95,100]. Langhoff et al. [104] noted that castrated piglets performed more tail wagging. Tail wagging was also observed by Lackner [105]; however, it was not considered a pain-specific behavior, but rather a way to drive away flies or occurring in combination with exploratory behavior. In contrast, Viscardi and Turner [98,99] observed more tail wagging and more pain-specific behaviors in castrated (saline-treated) piglets than in sham-castrated piglets; this also occurred 24 h after castration and was assumed to be due to inflammatory processes [98,99]. On the other hand, Yun et al. [69] claimed that tail wagging was detected less in castrated piglets than in non-castrated piglets, suggesting that it might also be related to positive emotions.

Taylor et al. [89] found that castrated piglets spend more time sitting or standing inactively in the first 2 h post-procedure; lying was further reduced in the next 22 h that followed. Conversely, Byrd et al. [106] found no differences with regard to lying in their treatment groups. Sutherland et al. [93], Sutherland et al. [87], and Sutherland et al. [95] also found that castrated piglets spent more time lying without contact with littermates. This observation was consistent with the findings of Viscardi and Turner [99], who reported more isolated castrated piglets at almost all time points within 24 h after castration. This might be, as summarized by Sutherland et al. [87], a protective behavior to avoid littermates and potentially painful situations, but could have negative consequences, such as temperature loss. Huddling up, a relieving posture to prevent pain due to tissue stretching [107], was more often observed in castrated piglets [102]. Castrated piglets were also found to display more pain-specific postures than handled piglets in the first hour after castration [9]. Davis et al. [108] evaluated the effect of castration on navigation through a chute, wherein it was found that castrated piglets needed more time to navigate through the chute when compared to the sham-castrated piglets.

An increase of blood or saliva cortisol has also been determined to be a common result of stressful situations, such as castration, and thus frequently reported [80]. These results were confirmed by Mühlbauer [109], Sutherland et al. [93], Sutherland et al. [87], Übel et al. [110], Sutherland et al. [95], Hofmann et al. [111], and Byrd et al. [106], wherein significantly increased cortisol concentrations in castrated piglets were observed at several time points after castration. Pain appeared to last for a couple of hours, as cortisol concentrations were back to basal values at 1 day post-procedure [109]. Cortisol was elevated in both handled and castrated piglets, but the increase was significantly higher in the castrated group, with a faster return to basal values [52,94]. Similar results were demonstrated by Lecchi et al. [76] upon analysis of salivary cortisol values, in which only the increase in sham-castrated piglets was insignificant. ACTH, cortisol, and lactate levels were elevated after up to 30, 60, and 90 min, respectively, indicating stress, pain, and tissue damage following castration [52]. Cortisol levels were also elevated from 60 min to more than 4 h after castration in another study (Zöls et al. [112]), but with no measurable difference after 28 h. Hay et al. [80] found higher cortisone levels on the first day post-procedure (tendency), but no difference in cortisol concentrations, as was also reported by Sutherland et al. [87] and Carroll et al. [101], however, it has to be kept in mind that the research design differed in these studies.

Heart and respiration rates were observed to increase more when the spermatic cords were ligated and cut than when the scrotum was incised and the testicles retrieved; however, heart rates decreased within 3 min after the procedure, supporting the finding that severing is the most stressful event [86]. Furthermore, the surface temperature of the skin reduced shortly after the procedure, in addition to a significantly increased glucose concentration in castrated pigs when compared to sham-castrated pigs, which was associated with the stress caused by castration [113]. On the other hand, Sutherland et al. [93] and Sutherland et al. [95] did not find differences between treatments with regard to total white blood cell counts and various leukocyte counts. CRP (C-reactive protein) concentrations tended to be greater in castrated piglets, which could be a sign of infection or inflammation, but there was no difference in substance P concentrations, which were measured to further evaluate nociception [87].

In suckling piglets, castration wounds are small with smooth margins that usually heal quickly without complications [114]. Swelling of the area around the wounds has been observed in the days after castration [100]. In addition to the pain caused by castration, piglets are often exposed to an increased risk of infection after the procedure [13]. However, Kluivers-Poodt et al. [103] reported fast healing with no signs of inflammation in castration wounds at the time of weaning. Lomax et al. [115] stimulated castration wounds and the surrounding tissue with von Frey weights and needles to test wound sensitivity: castrated piglets had the greatest response scores at all tested time points, whereas sham-castrated piglets and piglets receiving topical anesthesia were least likely to respond within 4 h after the procedure.

Neither weight gain nor survival rate appear to be influenced by castration, although handled control pigs have been shown to nurse longer than 2 week-old castrated pigs in the first 3 h after the procedure [116]. Since there was no measurable suppression in the feeding and drinking behavior of 7 week-old piglets up to 8 h after castration, castration was shown to have no long-lasting effect on production parameters. However, in an additional study, feeding time and weight gain were reduced in 8 week-old piglets [117]. Several authors could not detect differences in weight between study groups [80,87,93,94,95,103,113], nor differences in daily weight gain [110,114]. In several studies, no differences were noted in terms of mortality rates between treatment groups [94,111,113,116,117].

### 6.2. Effects of Alternative Castration Techniques

There are only a few studies comparing different castration techniques with often unclear results. Hay et al. [80] assumed that there may be long-term effects that have not yet been observed. Marchant-Forde et al. [13] noted that tearing of the spermatic cords required more time, hence significantly increasing the stress-related responses in the form of vocalizations and altered behavior; this was confirmed by a later study conducted by the same group [13], which explained that the gripping and pulling required during the tearing technique necessitated greater attention. The effects of cutting and tearing could not be clearly distinguished based on vocalizations [86]. The authors thus assumed that both severing methods are similarly painful, or that both procedures result in a maximum vocal response, which was confirmed by Marchant-Forde et al. [13].

When comparing the physical reactions of piglets castrated via tearing or cutting, cortisol concentrations were elevated 45 min after the procedure in both castrated groups [13], but increased β-endorphin values were only detected in piglets castrated by cutting, which may have been due to more bleeding, which was not quantified [13]. Furthermore, blood pH values decreased immediately after castration in both castrated and sham-castrated piglets [118]; this occurred independently of the number of incisions; however, the effect was more intense in piglets castrated with one incision rather with two. Lactate has also been shown to increase in both groups immediately after surgery [118]; these findings apparently interact and are assumed to have occurred due to damage to the innervated tissue during castration, thus inducing pain, but with a return to basal values within 6 h after castration.

According to EFSA [78], “castration is painful, regardless of the surgical procedure.” However, some castration techniques appear to be more painful than others, according to several study results and as suggested by Weary et al. [85]. Clamping with an emasculator limits bleeding [81], but increases the stress reaction [119]. Tearing of the cords is also believed to reduce bleeding; however, it can also produce a more ragged cut that may delay healing [80]. There were no differences in terms of wound healing among the piglets castrated by tearing or cutting [8]. Marchant-Forde et al. [13] also measured piglet body weight 1 and 2 weeks after processing: piglets whose spermatic cords were severed by tearing tended to show reduced growth rates when compared to sham piglets, but there was no difference between piglets castrated by cutting and control piglets [13].

### 6.3. Effect of Piglet Age at Castration

It has been generally assumed that neonatal piglets experience less pain; therefore, processing usually occurs at a young age [89]. Nevertheless, research has shown that castration is painful regardless of the age of the piglet [13,81,113]. As summarized by EFSA [78], there is no clear indication for lower pain in piglets younger than 1 week of age, and there may be more serious consequences for piglets castrated in the first 3 days after birth. Fredriksen et al. [4] found that castration usually takes place 3–7 days after birth, which is consistent with the results of the studies included in this present review (Figure 3 and Supplementary Table S4). Taylor et al. [89] noted higher call rates in piglets castrated at three different ages when compared to sham piglets, indicating a similar effect of castration on the immediate pain experienced by different age groups. This was in accordance with the results of White et al. [84], leading to the conclusion that pigs younger than 1 week old seem to experience as much pain as 2 or 3 week-old piglets [89].

Furthermore, piglets castrated at 2 and 7 weeks old both have demonstrated increased lying duration [116]. Moreover, piglets at 2 weeks of age spend more time lying and standing away from the heat lamp in the first 3 h after castration [116]. These findings were again observed in a later study with different aged piglets (1 to 20 days of age; [117]. Carroll et al. [101] found that piglets castrated at 3 days old stood more in the first hours after castration than those who were castrated as older piglets. McGlone and Hellman [116] found that both 2 and 7 week-old piglets experienced pain during castration, and all showed signs of their behavior being affected, but the effect was of a longer duration in the older age group; therefore, they assumed that sensitivity is reduced in younger piglets. When comparing the behavior of piglets castrated at 1 to 20 days, similar behavioral changes were observed [117]. This was confirmed by Taylor et al. [89] and Lackner [105], who also observed no age effect in terms of pain, concluding that castration at a younger age does not reduce pain. In fact, Prunier et al. [81] claimed that neonates might be even more sensitive to nociceptive stimuli than older individuals, due to their less-developed mechanisms for suppressing nociception.

In both 4 and 28 day-old piglets, adrenaline and noradrenaline concentrations were enhanced after handling, with the values increasing further for piglets castrated at 4 days old [105]. One day after castration, CRP, haptoglobin, and leukocyte counts increased in piglets castrated at both 4 and 28 days, but decreased in the following days. In the older age group, these parameters rose again after the third day, indicating continuous inflammatory processes and wound healing disorders [105]. In terms of the piglets’ immune response, it is recommended that castration be performed when the piglet is younger than 10 days of age, as antibody response and lymphocyte proliferation are disrupted in older piglets [120]; however, these authors were unsure whether castration at 3 days old has an impact on immunity at a later age. Cortisol concentrations at castration were not affected by whether the procedure was performed at 3, 6, 9, or 12 days of age [101]. However, after 48 h, cortisol was still elevated in those castrated at 6, 9, and 12 days old, as well as in control piglets. Thus, castration is stressful regardless of the age at which it is performed, but handling stress has been found to increase with age [101].

Lackner [105] noted better and faster healing after castration at 4 days old when compared to at 28 days old. When testing for wound sensitivity, smaller piglets showed the greatest probability of higher response scores, whereas larger piglets were least likely to respond [115]. With regard to growth, weight gain during lactation was determined to be higher in piglets castrated on day 14 when compared to piglets castrated on the first day after birth, but mortality was similar [117,121]. In addition, piglets castrated at 3 days old gained less weight than control animals, but this was not the case for those castrated at 10 days old [121]. The weight reduction in piglets castrated at a younger age may be due to a disadvantage during teat competition [81]. Nonetheless, Kielly et al. [121] and Lessard et al. [120] found no difference in terms of body weight between different age groups at the time of weaning.

### 6.4. Summary of Castration Effects and Practical Implications

Castration seems to be the most extensively studied routine procedure performed on young piglets (Figure 2). Many studies have, for example, evaluated the effect of castration on vocalizations, wherein most authors agree that the procedure alters vocalizations, such that they become higher in frequency and intensity. Behavioral changes are also well described: struggling, leg stretching, or increased lying during or in the first hours after castration are frequently listed as behaviors resulting from the pain and stress brought about by the procedure. Most of these behaviors seem to be transient and usually disappear after a few hours. Nonetheless, according to some studies, after some time, behaviors change in ways that could indicate longer-lasting pain. Several studies have also focused on cortisol concentrations; results are predominately consistent, with increased levels at various time points after castration. Weight development does not seem to be influenced by castration.

With respect to the different castration techniques, tearing takes a longer time, which causes more distress. However, according to the majority of behavior studies, vocalizations and cortisol concentrations are consistent, no matter what technique was used. It is assumed that castration causes a maximal pain response regardless of the applied technique. Furthermore, castration does not seem to affect weight development after the procedure, as there is only a tendency for reduced weight after tearing.

Additionally, with respect to age at castration, there are no clear effects: vocalizations and lying behavior are similar in piglets castrated at different ages, indicating that castration at a younger age does not—contradictory to common belief—lessen the pain that is experienced. Although there are signs of higher sensitivity to handling stress in older piglets, the temporary reduction in weight gain of piglets castrated at a very young age indicates that these piglets have a harder time handling the stress brought about by castration. Particularly, with respect to the increasing use of anesthesia for castration, one should consider that older piglets have a faster recovery rate and are better able to cope with food deprivation.

## 7. Interactions between and Comparison of Procedures

Studies examining the impacts of management procedures on the well-being of newborn piglets have predominately focused on castration, tail docking, and tooth clipping, which means that there is comparably less information available on the various identification techniques with respect to animal welfare [2,5]. Although some studies have explored the implementation of different identification techniques, as shown in Figure 3, these have mostly focused on the retention rate and readability of the technology, rather than on animal welfare, which was often only addressed in a side note. This may be explained by the legal obligation for pigs to be identified.

**Figure 4:**
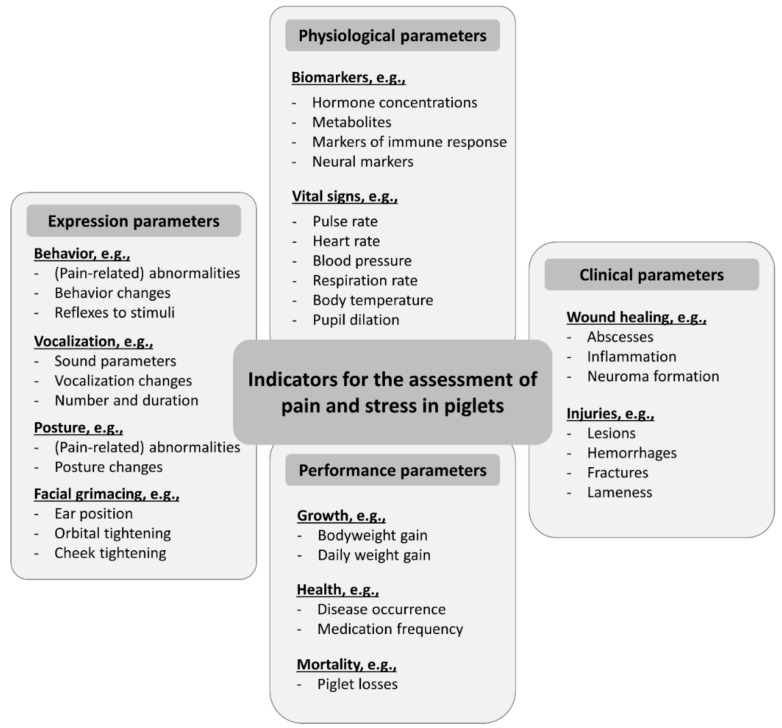
Summary of frequently used indicators for the assessment of pain and stress in piglets.

The groups of influencing indicators, i.e., welfare indicators, which have been used in the various studies investigated for this review, are introduced in Figure 2. A more detailed overview on the applied indicators is given in Figure 4. Here, the broad variety of indicators applied in studies investigating the effects of the discussed processing techniques on piglets are summarized.

**Figure 5:**
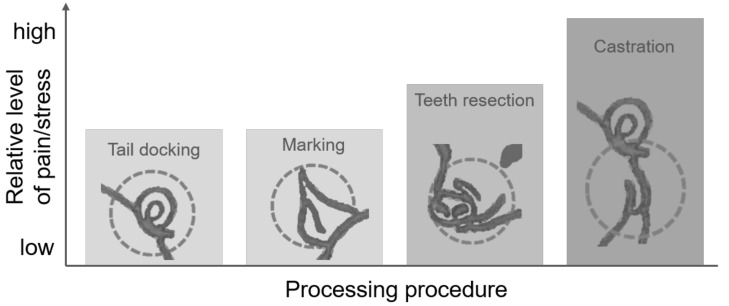
Relative pain and stress levels associated with four routine processing procedures: tail docking, marking for identification, teeth resection, and castration.

Castration has been found to be more painful and stressful than the other piglet processing events [2,9,10,13,52], inducing the highest pain level relative to all other invasive procedures [122], as is illustrated in Figure 5. Unlike tail docking or ear tagging, castration consists of several consecutive painful events (i.e., the initial incision of the scrotum, extraction of the testes, and severing of the spermatic cords [89]). Prunier et al. [81] emphasized that the scrotum, testes, and tissues associated with castration are highly innervated; therefore, any tissue damage in this area is likely to cause pain. During castration, the most painful moment appears to be the pulling and severing of the spermatic cords, as based on vocalizations [89,98] and resistance [123].

In comparison to castration, teeth resection or tail docking do not induce significant stress or pain [52]. Morrison and Hemsworth [124] observed lower vocalizations, escape attempts and cortisol values in tail-docked piglets than in castrated piglets. When compared to other procedures, such as castration, teeth resection, or ear notching, tail docking has been identified to have the least detrimental effect [2,9,38]. Then again, Cordeiro et al. [10] noted a lower vocalization intensity and duration in ear-notched piglets than in tail-docked and castrated piglets. When comparing procedures, it is important to remember that study results are obtained through the application of different techniques in piglets of varying ages (Supplementary Tables S1–S4). Furthermore, the effects of processing are often traced back to one specific procedure, although in some studies, other treatments (such as marking, weighing, or blood sampling) were simultaneously performed (Supplementary Tables S1–S4). This could complicate the reliability of the assessment and comparisons of their effects.

Every incidence of handling can induce stress in piglets, and many authors have reported signs of distress in handled and restrained piglets e.g., [5,13,40]. This raises the question of whether it is reasonable to combine routine processing procedures into one act in order to reduce handling; this could take place around the third day of life, as most procedures are performed at this time (Figure 3). Combining procedures may, however, result in cumulative stress [7], which could “mask” the effect of the single procedures [8]. Additionally, Übel et al. [110] found that a combined procedure increases distress in piglets when compared to one event (castration) alone. However, more research is needed to determine whether cumulative stress or single stress events are preferable in terms of animal welfare. In Germany and several other European countries, piglets must now be castrated under anesthesia and analgesia; thus, it would make sense to perform any other necessary procedures, such as ear tagging, when piglets are already anesthetized and have been administered pain relievers.

## 8. Conclusions

As there is a legal obligation to identify pigs, this procedure cannot be avoided. Thus, more research is needed to address the numerous research gaps identified in this review in order to minimize the negative effects of identification procedures. On the other hand, there is no legal requirement to perform teeth resection or tail docking; if anything, these procedures should only be performed in exceptional cases. Several studies with contradictory findings could not generate clear proof for the necessity of these interventions, indicating that their omission can be made possible. Therefore, rather than conducting additional studies on the impact of teeth resection and tail docking, the focus should shift to preventive measures, the management of problematic herds, and the investigation of practical solutions to avoid behaviors such as tail biting. A large number of studies have unmistakably demonstrated that surgical castration is the most painful intervention performed to piglets. Although its prevention has been discussed for years, it is likely that this practice will be continued for an indefinite period. Many authors have therefore focused on analgesia and anesthesia for castration, which is extremely essential. In addition to pain mitigation, adequate training of stockpersons, as well as their skill levels, has been identified as an important factor that would ensure a fast and accurate procedure to avoid further distress. Moreover, unavoidable castration should be performed using the least disturbing technique at the most resistant age. For this, more research and especially knowledge transfer to farmers is necessary to ensure appropriate implementation.

Generally, it must be presumed that invasive procedures impair welfare, even when no signs of distress are detected. The absence of any indices could result from the immense ability of piglets to compensate for their stress and impaired welfare; however, this does not justify humans taking advantage of it.

## Figures and Tables

**Table 1:** Frequently used identification methods for pigs and related tissue damages, readability, and retention rate. For information on other health implications of the respective identification systems and comparisons between techniques, consult Supplementary Table S1. Since the focus of this review is on suckling piglets, several studies investigating the identification of older pigs were excluded here. (TAB = transponder auricle base; TP = transponder perineum; TIP = transponder peritoneum).

Identification Method ^a^	Marking Site(s)	Tools Needed for Marking	Induced Tissue Damage	Readability	Losses	References
Ear tagging	Ear (one)					
Visual ear tag		Ear tag pliers, visual ear tag	Single punched hole	66–100% (on farm); 69–100% (at slaughter)	0–29% (on farm); 3–31% (at slaughter)	[16,17,18,19,20,21]
Electronic ear tag		Ear tag pliers, electronic ear tag		0–100% (on farm); 45–91% (at slaughter)	0–45% (on farm); 0–31% (at slaughter)	[16,17,18,20,21,22,23]
Ear notching	Ear (both)	Ear notching pliers	Multiple notched marks	-	-	-
Tattooing	Ear (one)	Tattoo pliers, character dies, ink	Multiple dies punctures, injected ink	0–56.3% (on farm) 0% (at slaughter)	-	[18,20,22]
Microchipping	Auricle base (TAB), perineum (TP), peritoneum (TIP)	Syringe and needle, transponder	Single needle puncture, injected transponder	TAB: 22.5–100% (on farm); 18–98% (at slaughter) TP: 100% (at slaughter) TIP: 69–100% (on farm); 69–100% (at slaughter) ^b^	TAB: 17–73 (on farm); 10% (at slaughter) TIP: 0–2% (on farm) ^b^	[16,17,18,19,21,22,23,24]

^a^ This table contains only identification techniques performed in the early stages of a pig’s life; therefore, the slap-marking of slaughter pigs was excluded. ^b^ Microchip readability decreases, and losses increase with transponder size.

## Data Availability

All data generated or analyzed during this study are included in this published article and its supplementary information files.

## Supplementary Materials

The following are available online at https://www.mdpi.com/article/10.3390/vetsci9010032/s1, Table S1: Overview of peer-reviewed studies investigating the effect of marking for identification in suckling piglets, Table S2: Overview of peer-reviewed studies investigating the effect of teeth resection in suckling piglets, Table S3: Overview of peer-reviewed studies investigating the effect of tail docking in suckling piglets, Table S4: Overview of peer-reviewed studies investigating the effect of castration in suckling piglets.
