# Supplementary material for: Impact of Routine Management Procedures on the Welfare of Suckling Piglets
Source: Vet Sci. 2022 Jan 17;9(1):32. doi: 10.3390/vetsci9010032 (PMC8778417; doi:10.3390/vetsci9010032)
Supplement: Supplementary file 1 [file vetsci-09-00032-s001.zip › vetsci-1508678-supplementary.pdf]

veterinary sciences

MDPI

Review

Impact of Routine Management Procedures on the Welfare of Suckling Piglets

Simone M. Schmid and Julia Steinhoff-Wagner

Supplementary Materials:

Table S1. Overview of peer-reviewed studies investigating the effect of marking for identification in suckling piglets.

Author (year)	Setting/ Groups (n)	Piglet age	Technique(s) (n)	Other procedures performed (n)	Investigated parameters				Main findings
					B&V	PP	WH	G&V	
Babot *et al.* (2006)	ID (1822)	1-3 w	ET (1533); EET (1446); TIP (1455)	ID with 2 or 3 devices			X	X	**Short- and long-term**<br>-No negative effects on animal welfare and performance<br>-No relevant infections or inflammatory reactions after ET
Barbieri *et al.* (2012)	ID (96)	10 d	EET, TAB, TIP, T	-	X		X		**Acute**<br>-Time required for application differed ($p < 0.001$)<br>**Short- and long-term**<br>-No healing problems or breakages of the electronic devices<br>-Most frequent behaviors: head shaking and ear scratching; more in piglets after EET ($p < 0.001$)
Bergqvist *et al.* (2015)	ID (80)	4 d (EET); 1–2 w and 9–10 w (TAB)	EET, TAB (different sizes)	-			X	X	**Short- and long-term**<br>-TAB better at 9–10 weeks old (retention rate; $p = 0.058$)<br>-ET more tissue damage than TAB ($p = 0.001$); lesions at slaughter
Bovey *et al.* (2014)	ID < 1 kg (40), ID ≥ 1.2 kg (80)	1 d (60), 3 d (60)	EN	TD (COLD), BS	X	X		X	**Acute**<br>-Light piglets fewer calls ($p < 0.05$), heavier piglets processed on d 3 higher frequency ($p = 0.05$)<br>**Short- and long-term**<br>-More dog-sitting, less lying, more isolation, less sucking (males) in light group ($p < 0.01$)

Author (year)	Setting/ Groups (n)	Piglet age	Technique(s) (n)	Other procedures performed (n)	Investigated parameters B&V	PP	WH	G&V	Main findings
									-Immunoglobulins lower in light group regardless of age at processing ($p \leq 0.06$)<br>-Higher mortality in light piglets ($p < 0.01$)
Caja *et al.* (2005)	ID (557)	0 d	ET (348), EET (209), TIP (309), TAB (248) (different sizes and devices)	-			X		**Acute**<br>-Time required for TIP and TAB differed ($p < 0.001$)<br>**Short- and long-term**<br>-No apparent animal health alterations observed<br>-After TIP, no inflammatory reactions or abscesses<br>-No reactions after ET and EET<br>-No effect of TIP on growth
Cordeiro *et al.* (2018)	ID (20)	7 d	EN	TD (HOT), CAS (PULL)	X				**Acute**<br>-Lower signal intensity during handling than during EN, TD, CAS<br>-Signal duration increased from with handling, EN, TD, CAS
Gosálvez *et al.* (2007)	ID (351)	35 d, 124 d	ET & T (left ear), EET (right ear)	-			X	X	**Short- and long-term**<br>-No infections, inflammatory reactions, or alterations in ear or injection area<br>-No negative effects on pig growth
Gruys *et al.* (1993)	ID (55)	3 w	TAB (glass-encapsulated)	-			X		**Short- and long-term**<br>-No abscesses found, no foreign body reaction<br>-Signs of inflammation after 3 d
Lambooij *et al.* (1992)	ID (56)	4–5 w	TAB (PET-covered), injection with different needles	-			X		**Short- and long-term**<br>-From d 2 inflammatory signs in injection areas, decrease after d 3<br>-2nd inflammation series from day 7, continuous swelling
Lambooij *et al.* (1995)	ID (5947)	4–5 w	TAB (different devices)	-			X		**Short- and long-term**<br>-3 weeks after injection: inflammation in 0.6% piglets
Lammers *et al.* (1995)	ID (204)	10 d, 4 w, 6 m	TAB	-			X		**Acute**<br>-Difficult injection in 10 d pigs, easier at 4 w<br>-Exudate around transponder only in 1 gilt

Author (year)	Setting/ Groups (n)	Piglet age	Technique(s) (n)	Other procedures performed (n)	Investigated parameters				Main findings
					B&V	PP	WH	G&V	
Leslie *et al.* (2010)	ID, SHAMear, SHAMTIP (total: 120)	4–12 d	ET, EN, TIP (glass)	SS, BS	X	X		X	**Acute**<br>-Highest sound pressure among EN ($p = 0.059$)<br>-Lactate levels increased ($p < 0.05$) in ear notched animals<br>-Cortisol increased ($p < 0.05$); no differences between groups<br>**Short-term**<br>-Increase in awake inactive behaviors relative to controls<br>-Longer periods of isolation in ET and TIP injected piglets ($p < 0.05$)<br>-Isolation among ET not different from EN; higher ($p < 0.05$) than SHAMear and SHAMTIP
Llamas Moya *et al.* (2007)	ID, CON (total: 96 piglets)	0 d	EN	TR, TD, BS		X			**Short-term**<br>-TR: tended to have higher levels of plasma Haptoglobin than CON, no other differences<br>-age effects were not affected by processing; no systemic inflammation in early life due to processing
Lomax *et al.* (2018)	ID (10), SHAM (10)	3–5 d	EN	-	X				**Acute**<br>-Probability to respond to EN: 98.7% for EN piglets, 0.9% for SHAM
Marchant-Forde *et al.* (2009)	ID (32), SHAM + blood (16), SHAM (16)	2–3 d	EN (16), ET (16)	BS	X	X	X	X	**Acute**<br>-EN longer duration than ET<br>-EN: calls with higher peak frequencies than SHAM groups<br>**Short- and long-term**<br>-EN: worse wound scores than ET ($p < 0.05$)<br>-Cortisol at 4 h greater in EN ($p < 0.10$)
Marchant-Forde *et al.* (2014)	ID (40), SHAM + blood (20), SHAM (20)	2–3 d	ET (20), EN (20)	TR (CLIP or GRIND), IA (INJ or ORAL), CAS (CUT or PULL), TD	X	X	X	X	**Acute**<br>-EN longer duration<br>-EN and ET more vocalization, escape attempts than SHAM ($p < 0.05$)<br>**Short- and long-term**<br>-Cortisol higher after 45 minutes in ET and EN ($p < 0.05$)

Author (year)	Setting/ Groups (n)	Piglet age	Technique(s) (n)	Other procedures performed (n)	Investigated parameters				Main findings
					B&V	PP	WH	G&V	
				(HOT or COLD), BS					-1 w after EN higher ß-endorphin, more ear wounds ($p < 0.05$)
Noonan *et al.* (1994)	ID (111), SHAM (56)	1–3 d	EN	TD (56), TR (55)	X				**Short-term**<br>-Processed piglets: different behavior ($p < 0.05$); head shaking after EN<br>-Restraint also stressful<br>-No age effect
Numberger *et al.* (2016)	ID (30), SHAM (30), a. o.	3–4 d	ET	BS		X			**Short-term**<br>-At 7 h cortisol returned to base values in all groups<br>-ET: greater cortisol response than SHAM at 30 and 60 min ($p \leq 0.001$)
Prola *et al.* (2010)	ID (60)	6–10 d	ET (15), TIP (15), TAB (15), TP (15)	-			X		**Acute**<br>-No apparent disturbances at application<br>**Short- and long-term**<br>-After TIP and ET, no inflammatory reactions or abscesses<br>-No health problems observed
Stärk *et al.* (1998)	ID (180)	3–4 w	ET, EET, TAB (2 devices)	-			X		**Acute**<br>-Use of single shot needles more time consuming<br>**Short- and long-term**<br>-Signs of infection at implantation site in 3.3% of pigs (only type A)<br>-Faster healing, less swelling, fewer infections with product B
Torrey *et al.* (2009)	ID (40), SHAM (40), CON (40)	1 d, 3 d	EN	TD, BS	X	X		X	**Acute**<br>-EN, regardless of age, greater frequency ($p < 0.001$) and more high frequency calls ($p = 0.016$) than SHAM<br>-D 1: more high frequency calls than on d 3 ($p = 0.047$)<br>**Short-term**<br>-SHAM and EN: less time lying and more time standing than CON ($p < 0.001$)<br>-EN: more tail jamming than SHAM or CON ($p < 0.001$)

Author (year)	Setting/ Groups (n)	Piglet age	Technique(s) (n)	Other procedures performed (n)	Investigated parameters				Main findings
					B&V	PP	WH	G&V	
									-Lying, standing and tail posture not influenced by age<br>-More trembling in d 1 piglets than in piglets on d 3 ($p < 0.001$)

a. o. = and others; BS = blood sampling; B&V = Behavior & vocalization; CAS = castration; CON = control piglets (not handled); d = days; EET = electronic ear tag; EN = ear notching; ET = ear tagging; G&V = growth & vitality; IA = iron application; ID = marking for identification; INJ = injection; m = months; PP = physiological parameters; SHAM = sham-handled piglets; SS = saliva sampling; T = tattoo; TAB = transponder at auricle base; TD = tail docking; TIP = intraperitoneal injected transponder; TP = transponder at perineum; TR = teeth resection; w = weeks; WH = wound healing

Table S2. Overview of peer-reviewed studies investigating the effect of teeth resection in suckling piglets.

Author (year)	Setting/ Groups (n)	Piglet age	Technique(s) (n)	Other procedures performed (n)	Investigated parameters				Main findings
					B&V	PP	WH	G&V	
Bates *et al.* (2002)	TR (1592), CON (1602)	0 d	CLIP	TD, EN, CAS, IA			X	X	**Long-term**<br>-Higher mortality ($p < 0.001$) in CLIP than in CON (parity 6+, trend in parity 1)<br>-Mortality not different between treatments in parity 2–5 females<br>-CON: higher ($p < 0.001$) face scores than CLIP
Boyle *et al.* (2002)	TR (12 litters), CON (12 litters)	0 d	CLIP	EN, TD	X			X	**Short- and long-term**<br>-CON: more walking/running/playing on d 5 and 15<br>-CLIP: longer sleeping than CON on d 15
Brookes and Lean (1993)	TR (20), CON (10)	0 d	CLIP (10), GRIND (10),	-	X		X	X	**Short- and long-term**<br>-No weight differences between treatments ($p > 0.05$)<br>-CON: more face wounds; least wounding in CLIP ($p < 0.01$)
Brown *et al.* (1996)	TR, SHAM (total: 550)	0 d	CLIP	BS	X		X	X	**Short- and long-term**<br>-Higher facial lesion scores at d 7, 14, 22 in CON<br>-No effect on survival or weight gain
Fraser and Thompson (1991)	TR (50), CON (42)	0 d	CLIP	-	X		X	X	**Short- and long-term**<br>-Facial wounding more in CON<br>-Wounding/fighting more among large litters ($p < 0.05$).<br>-No differences in weight gain to w 3<br>-No gross differences in development of suckling order
Fu *et al.* (2018)	TR (152), SHAM (150)	3 d	CLIP	TD (75), SHAMTD (77)		X		X	**Long-term**<br>-More social interactions in CLIP<br>-CON: higher respiration rate<br>-No effect on body weight and mortality between 30–85 d
Fu *et al.* (2019)	TR (151), SHAM (74)	3 d	CLIP	TD (75), SHAMTD (76)	X	X	X		**Acute**<br>-Heart rates increased ($p < 0.01$) in CLIP<br>-CLIP: decreased body surface temperature ($p < 0.01$)<br>**Short- and long-term**

Author (year)	Setting/ Groups (n)	Piglet age	Technique(s) (n)	Other procedures performed (n)	Investigated parameters B&V	PP	WH	G&V	Main findings
									-CLIP: more ($p < 0.05$) time lying alone and playing/fighting than SHAM; lower lesion scores on teats ($p < 0.05$)
Gallois *et al.* (2005)	TR (70 litters), CON (35 litters)	0 d	CLIP (35 litters), GRIND (35 litters)	-			X	X	**Long-term**<br>-Litter size and weight on d 0 and 27 similar<br>-Skin lesions: more frequent/severe in CON on d 8 and 27, intermediate in GRIND<br>-Similar teeth length in CLIP and GRIND ($p > 0.1$)
Hansson and Lundeheim (2012)	TR (28 litters), CON (36 litters)	0 d	GRIND	-			X	X	**Short- and long-term**<br>-No effect of treatment on facial lesion score<br>-Facial lesion scores higher in w 1 than in w 2 ($p < 0.001$), higher in large litters ($p = 0.003$)<br>-Mortality between w 1 and w 2 higher in CON ($p = 0.02$)
Hay *et al.* (2004)	TR/CON (20)	1–2 d	CLIP + GRIND + CON (all in each piglet)	-			X		**Short- and long-term**<br>-TR: lesions such as pulp cavity opening, fracture, hemorrhage, infiltration or abscess; sooner/more severe in CLIP
Hessling-Zeinen (2014)	TR (87)	0 d	GRIND	-			X		**Short-term**<br>-Open teeth in > 90% of examined animals<br>-45% of all pulp cavities opened<br>-GRIND: opening of pulp cavities in nearly all piglets<br>-Differences between farms ($p < 0{,}05$)
Holyoake *et al.* (2004)	TR, CON (total: 135 litters)	0 d	CLIP (71 litters), GRIND (64 litters)	-			X	X	**Short- and long-term**<br>-CLIP: higher weaning weight than GRIND; not than CON<br>-CLIP: fewer preweaning deaths than both CON and GRIND (fewer overlays)<br>-Higher incidence/severity of face wounds in CON than CLIP/GROUND<br>-No treatment effect on face scars at weaning, udder damage, deaths or weight gain
Hutter *et al.* (1993)	TR (490), CON (306)	0 d	CLIP (182), GRIND (308)	-			X	X	**Short- and long-term**<br>-48% of GRIND teeth with inflammation

Author (year)	Setting/ Groups (n)	Piglet age	Technique(s) (n)	Other procedures performed (n)	Investigated parameters				Main findings
					B&V	PP	WH	G&V	
									-CLIP: pulpitis in 92% of teeth; inflammation of gingiva; splinters<br>-Bites more frequent in CON than in TR<br>-Lowest mortality in CLIP
Kober and Thacker (1999)	TR (84 litters)	1 d (43 litters), 2 d (41 litters)	CLIP	TD, IA				X	**Long-term**<br>-Mortality lower for d 1-piglets ($p < 0.05$)<br>Litter and piglet weight higher in d 1-piglets at weaning ($p < 0.05$)
Lewis *et al.* (2005)	TR (40 litters), CON (20 litters)	0 d	CLIP (20 litters), GRIND (20 litters)	EN	X		X	X	**Acute**<br>-CLIP more chomping than CON after 5 min ($p = 0.05$)<br>**Short-term**<br>-After 30 min CON more active than TR ($p < 0.05$)<br>**Long-term**<br>-D 21: CON more active than GRIND ($p < 0.05$)<br>-D 26: GRIND more inactive than CON and CLIP ($p < 0.05$)<br>-D 14/26, CLIP more sleeping than GRIND ($p = 0.05$)
Llamas Moya *et al.* (2006)	TR, CON (total: 60 litters)	0 d	CLIP, GRIND	EN, BS		X	X		**Short-term**<br>-No difference in C reactive protein/serum amyloid A on d 1<br>**Long-term**<br>-Concentrations higher on d 29<br>-CLIP: higher C reactive protein than GRIND on d 29
Llamas Moya *et al.* (2007)	TR, CON (total: 96 piglets)	0 d	CLIP	EN, TD, BS		X			**Short-term**<br>-TR: tended to have higher levels of plasma Haptoglobin than CON, no other differences<br>-age effects were not affected by processing; no systemic inflammation in early life due to processing
Marchant-Forde *et al.* (2009)	TR (32), SHAM + blood (16), SHAM (16)	2–3 d	CLIP (16), GRIND (16)	BS	X	X	X	X	**Acute**<br>-GRIND longer duration than CLIP; greater cortisol concentration<br>-longer vocalizations than SHAM groups ($p < 0.05$)

Author (year)	Setting/ Groups (n)	Piglet age	Technique(s) (n)	Other procedures performed (n)	Investigated parameters				Main findings
					B&V	PP	WH	G&V	
									**Short- and long-term**<br>-TR: poorer growth than SHAM groups ($p < 0.05$)
Marchant-Forde *et al.* (2014)	TR (40), SHAM + blood (20), SHAM (20)	2–3 d	CLIP (20), GRIND (20)	ID (EN or ET), IA (INJ or ORAL), CAS (CUT or PULL), TD (HOT or COLD), BS	X	X	X	X	**Acute**<br>-TR longer duration than SHAM; more vocalizations, escape attempts ($p < 0.05$)<br>**Short- and long-term**<br>-Cortisol after 45 min higher in TR than in SHAM groups ($p < 0.05$)<br>-More Stressful took longer; higher β-endorphin at w 1<br>-Growth during d 2 to 7 lower in More Stressful than SHAM ($p < 0.05$); by w 2 no effect
Menegatti *et al.* (2018)	TR, CON (total: 15 litters)	0 d	CLIP, GRIND	-		X	X	X	**Short-term**<br>-W 1: GRIND higher weight gain<br>-After d 4: CLIP/GRIND reduced facial lesions<br>-No effect on blood serum protein<br>-W 2: CLIP worst weight gain<br>-W 1–2: CLIP worse weight gain than GRIND<br>-Mortality not influenced"
Meunier-Salaün *et al.* (2002)	TR, SHAM, CON (total: 152)	1 d	CLIP, GRIND	-	X	X			**Acute**<br>-GRIND: more leg movements<br>-More chewing behavior in TR<br>**Short- and long-term**<br>-D 1: similar resting, standing or activity at sow's udder similar<br>-D 7: more lip lesions in TR; CLIP lower weight than CON<br>-TR no effect on plasma cortisol, ACTH, glucose and lactate
Noonan *et al.* (1994)	TR (111), SHAM (56)	1–3 d	CLIP	EN (56), TD (55)	X				**Short-term**<br>-Processed piglets: different behavior ($p < 0.05$); CLIP: teeth champing<br>-Restraint also stressful<br>-No age effect

Author (year)	Setting/ Groups (n)	Piglet age	Technique(s) (n)	Other procedures performed (n)	Investigated parameters				Main findings
					B&V	PP	WH	G&V	
Prunier *et al.* (2005)	TR (13), SHAM (7), CON (7)	1 d	CLIP (6), GRIND (7)	BS		X			**Short-term**<br>-Plasma lactate higher in GRIND than in SHAM and CLIP, intermediate in CON<br>-TR no effect on plasma cortisol, ACTH, glucose, lactate
Robert *et al.* (1995)	TR, CON (total: 346 litters)	1 d	CLIP	EN				X	**Long-term**<br>-In large litters: lower-birth-weight piglets (CLIP) lower mortality<br>-Within-litter variance of 21-d weights smaller ($p < 0.00$) in CLIP
Sinclair *et al.* (2016)	TR, SHAM (total: 102)	1 d	CLIP, GRIND	-	X	X			**Acute**<br>-A treatment*time effect for standing ($p < 0.0001$), investigating ($p = 0.0152$)<br>**Short- and long-term**<br>-D 1: lower standing duration in GRIND, SHAM intermediate; higher inactivity in GRIND ($p = 0.003$)<br>-No effect for walking, oral behaviors, number of agnostic behaviors<br>-No effects on d 5, 12 or 26
Sinclair *et al.* (2018)	TR (32), SHAM (16)	-	CLIP (16), GRIND (16)	-			X		**Short- and long-term**<br>-All TR: pulp exposure; higher in CLIP<br>-CXCL8 gene expression increased ($p < 0.001$) in TR<br>-CALCB expression downregulated for CLIP ($p < 0.001$) and GRIND at w 1 ($p < 0.05$)
Sinclair *et al.* (2019)	TR (80), SHAM (40)	1 d	CLIP (40), GRIND (40)	-	X				**Acute**<br>-Minor bleeding after TR, higher in CLIP<br>**Short-term**<br>-Exploring higher in SHAM than in CLIP, GRIND intermediate<br>-Champing never before, but after TR different between SHAM and CLIP ($p < 0.05$), GRIND intermediate<br>-After TR: walking, exploring, ears back decreased ($p < 0.05$).

Author (year)	Setting/ Groups (n)	Piglet age	Technique(s) (n)	Other procedures performed (n)	Investigated parameters				Main findings
					B&V	PP	WH	G&V	
van Beirendonck *et al.* (2012)	TR (total: 251), CON (44)	1 d	GRIND	ET, TD, CAS, IA	X		X	X	**Short-term**<br>-Differences in behavior, but weight at weaning between GRIND and CON not different<br>-Mortality higher in lightest piglets, higher in GRIND
Weary and Fraser (1999)	TR, CON (total: 553)	0 d	CLIP partly, CLIP fully	-			X	X	**Short- and long-term**<br>-W 1: highest weight gain in CON, CLIP partly intermediate ($p < 0.02$); no effect on later weight, deaths, suckling position<br>-Facial lesions: negligible in partly and fully CLIP, greater in CON ($p < 0.02$)
Zhou *et al.* (2013)	TR (63), SHAM (63)	3 d	CLIP	TD	X	X	X	X	**Acute**<br>-CLIP more vocalization ($p < 0.01$)<br>**Short- and long-term**<br>-CLIP: more lying alone ($p = 0.03$) during entire suckling period<br>-CLIP did not ($p \geq 0.14$) alter suckling, standing, huddling, playing/fighting, sitting during d 1–3 and d 5–15<br>-CLIP: more resting ($p = 0.03$), less ($p \leq 0.01$) interested in exploratory behaviors; reduced weight gain between d 10–21 d ($p = 0.01$) and 21–70 d ($p = 0.04$)<br>-D 160 weight not affected by CLIP ($p = 0.62$); no difference between SHAM and CLIP for fat ($p \geq 0.05$), LM ($p = 0.93$), or muscle ($p = 0.27$)

a. o. = and others; BS = blood sampling; B&V = Behavior & vocalization; CAS = castration; CON = control piglets (not handled); d = days; EN = ear notching; G&V = growth & vitality; IA = iron application; INJ = injection; m = months; PP = physiological parameters; SHAM = sham-handled piglets; TD = tail docking; TR = teeth resection; w = weeks; WH = wound healing.

Table S3. Overview of peer-reviewed studies investigating the effect of tail docking in suckling piglets.

Author (year)	Setting/ Groups (n)	Piglet age	Technique(s) (n)	Other procedures performed (n)	Investigated parameters B&V	PP	WH	G&V	Main findings
Bovey *et al.* (2014)	TD < 1 kg (40), TD ≥ 1.2 kg (80)	1 d (60), 3 d (60)	COLD	EN, BS	X	X		X	**Acute**<br>-Light piglets fewer calls ($p < 0.05$), heavier piglets processed on d 3 higher frequency ($p = 0.05$)<br>**Short- and long-term**<br>-More dog-sitting, less lying, more isolation, less sucking (males) in light group ($p < 0.01$)<br>-Immunoglobulins lower in light group regardless of age at processing ($p \leq 0.06$)<br>-Higher mortality in light piglets ($p < 0.01$)
Cordeiro *et al.* (2018)	TD (20)	7 d	HOT	EN, CAS (PULL)	X				**Acute**<br>-Lower signal intensity during handling than during EN, TD, CAS<br>-Signal duration increased with handling, EN, TD, CAS
Di Giminiani *et al.* (2016)	TD (8)	3 d	HOT	-	X				**Short-term**<br>-TD: change ($p < 0.05$) only in the "orbital tightening" facial action unit
Di Giminiani *et al.* (2017)	TD, SHAM (total: 126)	3 d	HOT (10), COLD (10), TD long (24), short (24)	-	X		X		**Acute**<br>-Greater call energy and intensity in TD<br>**Short-term**<br>-No difference in activity up to 48 h post-procedure<br>**Long-term**<br>-No difference in mechanical nociceptive thresholds indicative of long-term pain observed at 17 w
Di Martino *et al.* (2015)	TD (128), CON (320)	5 d	HOT	CAS	X	X	X	X	**Long-term**<br>-TD no different blood parameters<br>-Higher cortisol in CON females than in TD females; higher cortisol in TD males than in CON males ($p < 0.04$)<br>-CON: more tail lesions; less belly nosing

Author (year)	Setting/ Groups (n)	Piglet age	Technique(s) (n)	Other procedures performed (n)	Investigated parameters				Main findings
					B&V	PP	WH	G&V	
									-CON: less explorative activity ($p < 0.05$); more lying behavior ($p < 0.02$).
Fu *et al.* (2018)	TD (151), SHAM (150)	3 d	-	TR (75), SHAMTR (74)		X		X	**Long-term**<br>-More social interactions in TD, fewer lesions on tail<br>-CON: higher respiration rate<br>-No effect on body weight and mortality between 30–85 d
Fu *et al.* (2019)	TD (152), SHAM TD/TR (74)	3 d	HOT	TR (75), SHAMTR (77)	X	X	X		**Acute**<br>-Heart rates increased ($p < 0.01$) during TD<br>-TD: no decreased body surface temperature<br>**Short- and long-term**<br>-TD: more ($p < 0.05$) time lying alone and playing/fighting than SHAM<br>-Lower impact than TR
Herskin *et al.* (2015)	TD (47), CON (18)	2–4 d	HOT; TD 75% (17), 50% (19), 25% (11) of tail	-			X		**Short and long-term**<br>-TD: more tails with neuromas, increased size and number of neuromas ($p < 0.001$)<br>-No neuroanatomical differences between docking lengths
Herskin *et al.* (2016)	TD (70), CON (57), a. o.	2–4 d	HOT; TD 75%, 50%, 25% of tail	-	X				**Short-term**<br>-TD: behavioural changes, persistent effects through 5 h<br>-More squealing, sudden jerking in TD than in SHAM<br>-Increased likelihood of squealing with increasing tail removal
Kober and Thacker (1999)	TD (84 litters)	1 d (43 litters), 2 d (41 litters)	-	TR (CLIP), IA				X	**Long-term**<br>-Mortality lower for d 1-piglets ($p < 0.05$)<br>-Litter and piglet weight higher in d 1-piglets at weaning ($p < 0.05$)
Llamas Moya *et al.* (2007)	TD, CON (total: 96 piglets)	0 d	-	EN, TR, BS		X			**Short-term**<br>-TR: tended to have higher levels of plasma Haptoglobin than CON, no other differences<br>-age effects were not affected by processing; no systemic inflammation in early life due to processing

Author (year)	Setting/ Groups (n)	Piglet age	Technique(s) (n)	Other procedures performed (n)	Investigated parameters				Main findings
					B&V	PP	WH	G&V	
Lecchi *et al.* (2020)	TD (12), SHAM (12), a. o.	4 d	HOT	CAS, IA, SS		X			**Short-term findings:**<br>-TD: salivary cortisol increased<br>-Abundance increase of miR-19b, miR-27b, miR-365 in saliva of TD<br>-No differences in SHAM
Marchant-Forde *et al.* (2009)	TD (32), SHAM + blood (16), SHAM (16)	2–3 d	HOT (16), COLD (16)	BS	X	X	X	X	**Acute**<br>-HOT longer duration than COLD; longer and higher frequency squealing compared with SHAM groups<br>**Short- and long-term**<br>-HOT: slower growth than COLD ($p < 0.01$)
Marchant-Forde *et al.* (2014)	TD (40), SHAM + blood (20), SHAM (20)	2–3 d	HOT (20), COLD (20)	ID (EN or ET), IA (INJ or ORAL), CAS (CUT or PULL), TR (CLIP or GRIND), BS	X	X	X	X	**Acute**<br>-TD longer duration than SHAM; more vocalizations, escape attempts ($p < 0.05$)<br>**Short- and long-term**<br>-Cortisol after 45 min higher in TD than in SHAM groups ($p < 0.05$)<br>-More Stressful (HOT) took longer; higher β-endorphin at w 1 and increased tail wound scores ($p < 0.01$)<br>-Growth during d 2 to 7 lower in More Stressful (HOT) than SHAM ($p < 0.05$); by w 2 no effect
Morrison and Hemsworth (2020a)	TD, SHAM, a. o. (total: 288)	2 d	HOT, COLD	BS	X	X			**Acute**<br>-Duration of vocalization and escape attempts greater during CAS than SHAM and TD<br>**Short-term**<br>-Higher cortisol after 15 min in TD and CAS<br>-30 min: cortisol only higher in COLD and CAS<br>-More pain behavior in TD and CAS at 60 min, but no treatment effect after 24 h
Morrison and Hemsworth (2020b)	TD, SHAM, CON, a. o. (total: 432)	2 d	HOT, COLD	BS	X	X			**Acute**<br>-Duration of vocalizations and frequency of escape attempts greater in all TD than SHAM<br>**Short-term**

Author (year)	Setting/ Groups (n)	Piglet age	Technique(s) (n)	Other procedures performed (n)	Investigated parameters				Main findings
					B&V	PP	WH	G&V	
									-COLD: higher cortisol at 30 min, but not at 15 min, and stood longer with lowered head
Noonan *et al.* (1994)	TD (111), SHAM (56)	1–3 d	-	EN (56), TR (56)	X				**Acute**<br>-More grunting during and immediately after procedure in TD<br>**Short-term**<br>-Processed piglets: different behavior ($p < 0.05$); TD caused more tail jamming and wagging<br>-Restraint also stressful<br>-No age effect
Numberger *et al.* (2016)	TD (30), SHAM (30), a. o.	3–4 d	COLD	BS		X			**Short-term**<br>-At 7 h cortisol back to base values in all groups<br>-ET and CAS greater response than TD<br>-TD: greater cortisol response than SHAM at 30 min
Prunier *et al.* (2001)	TD, SHAM, CON, a. o. (total: 180)	1 d	HOT	-	X	X		X	**Acute**<br>-During 20 s after TD, more tail jamming and wagging ($p < 0.05$)<br>**Short-term**<br>-During 12 h, resting/activity similar between groups<br>-Growth rate in 1st week and injuries at tail not different ($p > 0.1$)
Prunier *et al.* (2005)	TD, SHAM, CON (total: 17)	-	HOT	BS		X			**Short-term**<br>-No treatment × time interaction<br>-Glucose lower ($p < 0.05$) in SHAM than in TD and CON
Sandercock *et al.* (2011)	TD, SHAM, a. o. (total: 109)	2–4 d	COLD	-		X			**Long-term**<br>-Exp. 1 (8 w): no altered thresholds to mechanical stimulation after TD<br>-Exp. 2 (5 w): no altered thresholds to mechanical or cold stimulation after TD
Sandercock *et al.* (2016)	TD (4)	3 d	HOT	-			X		**Long-term**<br>-Non-neural inflammatory and reparative epidermal and dermal changes associated with tissue thickening and healing

Author (year)	Setting/ Groups (n)	Piglet age	Technique(s) (n)	Other procedures performed (n)	Investigated parameters				Main findings
					B&V	PP	WH	G&V	
									-Mild inflammation present in some; traumatic neuroma after 1 month, still incomplete after 4 months
Sandercock *et al.* (2019)	TD (48), SHAM (48)	3 d, 63 d	HOT	-		X	X	X	**Long-term**<br>-TD: changes in gene expression (up and down) compared to SHAM (all ages)<br>-Changes in gene expression in TD evident 4 months after tail injury<br>-No long-term alterations of nociception in pigs
Simonsen *et al.* (1991)	TD (30), CON (10)	1 d (TD), > 90 kg (TD+CAS), > 90 kg (CON)	COLD	-		X	X		**Short- and long-term**<br>-Both ages: peripheral nerves unevenly distributed, regressive changes; traumatic neuromas indicating increased sensitivity in stump
Simonsen (1995)	TD, CON (total: 576)	1 d	COLD	-	X	X			**Long-term**<br>-Tail status no effect on nibbling and tail biting
Sutherland *et al.* (2008)	TD (40), CON (40)	6 d	HOT (20), COLD (20)	BS	X	X			**Short-term**<br>-TD: more ($p < 0.07$) sitting than CON at 0–15 min<br>-White blood cells reduced ($p < 0.05$) in TD compared to CON at 30 min<br>-Cortisol: higher ($p < 0.01$) among COLD compared with CON and HOT at 60 min<br>-TD more ($p < 0.05$) scooting than CON at 0–15 min, 31–45 min
Sutherland *et al.* (2009)	TD (40), CON (40)	6 d	HOT (20), COLD (20) (Exp. 1); SHORT, LONG (Exp. 2)	BS		X	X		**Long-term**<br>-Tail-biting lesions similar at 3 and 5 weeks<br>-7 weeks: lesions greater among CON compared to TD<br>-Bodyweights lower in CON compared to TD<br>-C reactive protein elevated in CON compared to TD at 7 weeks<br>-Tail-biting lesions greater in LONG than in SHORT tails

Author (year)	Setting/ Groups (n)	Piglet age	Technique(s) (n)	Other procedures performed (n)	Investigated parameters				Main findings
					B&V	PP	WH	G&V	
Sutherland *et al.* (2011)	TD (20), SHAM (20), a. o.	3 d	COLD	BS	X	X	X	X	**Acute**<br>-Stress vocalizations increased in TD compared to TD<br>**Short-term**<br>-Neutrophil/lymphocyte ratio greater in TD than in SHAM<br>-Cortisol higher in CUT at 30 min<br>-More TD pigs lying without contact than SHAM<br>**Long-term**<br>-Body weight and wound scores did not differ between treatments
Tallet *et al.* (2019)	TD (48), SHAM (50), CON (47)	1–3 d	HOT	-	X			X	**Acute**<br>-TD more screaming, higher intensity ($p < 0.05$) than SHAM<br>-TD: ears perpendicular to the head-tail axis; more changes in ear posture ($p < 0.05$)
Torrey *et al.* (2009)	TD (40), SHAM (40), CON (40)	1 d, 3 d	COLD	EN, BS	X	X		X	**Acute**<br>-TD (all ages): greater frequency ($p < 0.001$) and more high frequency calls ($p = 0.016$) than SHAM<br>-D 1: more high frequency calls than on d 3 ($p = 0.047$)<br>**Short-term**<br>-SHAM and TD: less time lying and more time standing than CON ($p < 0.001$)<br>-TD: more tail jamming than SHAM or CON ($p < 0.001$)<br>-Lying, standing and tail posture not influenced by age<br>-More trembling in d 1 piglets than in piglets on d 3 ($p < 0.001$)
Übel *et al.* (2015)	TD, SHAM, a. o.	3–4 d	COLD	ET, CAS, IA, BS	X	X			**Short-term**<br>-Higher cortisol for 4 h after CAS + TD + ET than after CAS and SHAM<br>-Weights not different 4 d after SHAM and CAS + TD + ET
van Beirendonck *et al.* (2012)	TD (total: 251), CON (44)	1 d	-	ET, TR, IA	X		X	X	**Short- and long-term**<br>-Differences in behavior, but weight at weaning not different<br>-Mortality higher in lightest piglets

Author (year)	Setting/ Groups (n)	Piglet age	Technique(s) (n)	Other procedures performed (n)	Investigated parameters B&V	PP	WH	G&V	Main findings
Viscardi *et al.* (2017)	CAS + TD (2), CAS + TD + NaClA (5)	5 d	1 INC, PULL	TD (COLD), IA	X				**Short-term**<br>-More inactive behaviors up to 6 h post-CAS and TD<br>-Behavioral changes up to 7 h post-CAS compared to baseline<br>-Higher grimace scores at 0, 3, 4, 5 h post-CAS compared to scores at 7 h
Viscardi and Turner (2019)	TD (30), SHAM (15), a. o.	4 d	COLD	-	X				**Acute**<br>-TD: higher vocalizations than SHAM ($p < 0.05$)<br>**Short-term**<br>-More grimacing in TD than in SHAM ($p = 0.02$)
Vitali *et al.* (2020)	TD, CON	3–6 d	-	CAS	X				**Long-term**<br>-Tear staining higher in TD than in CON ($p = 0.05$)<br>-CON: higher Qualitative Behavior Assessment score than TD piglets ($p = 0.01$)<br>-Tail lesion score index not influenced by TD
Zhou *et al.* (2013)	TD (63), SHAM (63)	3 d	COLD	TR	X	X	X	X	**Acute**<br>-TD more vocalization ($p < 0.01$)<br>**Short- and long-term**<br>-TD: more lying alone ($p = 0.03$) during entire suckling period<br>-TD no alteration of ($p \geq 0.14$) suckling, standing, huddling, playing/fighting, sitting during d 1–3 and d 5–15<br>-TD: more resting ($p = 0.03$), less ($p \leq 0.01$) interested in exploratory behaviors; reduced weight gain between d 10–21 d ($p = 0.01$) and 21–70 d ($p = 0.04$)<br>-D 160 weight not affected by TD ($p = 0.62$); no difference between SHAM and TD for fat ($p \geq 0.05$), LM ($p = 0.93$), or muscle ($p = 0.27$)

a. o. = and others; BS = blood sampling; B&V = Behavior & vocalization; CAS = castration; CON = control piglets (not handled); COLD = cold tail docking (side cutters); d = days; EN = ear notching; ET = ear tagging; Exp. = experiment; G&V = growth & vitality; HOT = hot tail docking (cautery); IA = iron application; INJ = injection; m = months; PP = physiological parameters; SHAM = sham-handled piglets; SS = salivary sample; TD = tail docking; TR = teeth resection; w = weeks; WH = wound healing.

Table S4. Overview of peer-reviewed studies investigating the effect of castration in suckling piglets.

Author (year)	Setting/ Groups (n)	Piglet age (n)	Technique(s) (n)	Other procedures performed (n)	Investigated parameters B&V	PP	WH	G&V	Main findings
Abendschön *et al.* (2020)	CAS (12), SHAM (11), a. o.	3–7 d	2 INC, CUT	NaClA	X				**Acute**<br>-CAS: more defensive movements than SHAM during incision<br>-Highest score found in CAS during severing of cords, SHAM reduced movements and vocalizations ($p < 0.05$)<br>-No difference with regard to navigation time
Bonastre *et al.* (2016)	CAS (15), SHAM (15), a. o.	4–7 d	2 INC, CUT	-		X		X	**Acute**<br>-Decreased skin temperature in CAS<br>**Short-term**<br>-Higher cortisol in CAS<br>**Long-term**<br>-Bodyweight not different between groups
Byrd *et al.* (2020)	CAS + BS (8), CAS (7), SHAM + BS (7), SHAM (8)	10 d	1 INC, CUT	BS, HRV		X			**Short-term**<br>-CAS: greater low to high frequency ratio compared to SHAM<br>-Cortisol greater in CAS at 1 h compared to SHAM<br>-No effect of treatment lying behavior
Carroll *et al.* (2006)	CAS (45), SHAM (45)	3 d (23), 6 d (25), 9 d (24), 12 d (18)	2 INC, CUT	BS	X	X		X	**Acute**<br>-Cortisol greater in CAS than in SHAM<br>-No overall effect of age at castration on cortisol<br>**Short-term**<br>-At 2 h age effect ($p = 0.01$) on standing: 3 d-old pigs more than 6, 9, or 12 d-old pigs<br>-no effect on growth at 24 or 48 h<br>-cortisol back at baseline in all groups at 24 h; after 48 h elevated ($p < 0.01$) in 6, 9, and 12 d-old pigs in CAS and SHAM
Cordeiro *et al.* (2018)	CAS (20)	7 d	1 INC, PULL	TD (HOT), EN	X				**Acute**<br>-Lower signal intensity during handling than during EN, TD, CAS<br>-Signal duration increased with handling, EN, TD, CAS
Davis *et al.* (2017)	CAS (25), SHAM (26), a. o.	2–4 d	2 INC, PULL	WAO, BS	X	X			**Acute**<br>-At 0, 15 and 30 min: CAS greater navigation time than SHAM ($p < 0.05$)
Gottardo *et al.* (2016)	CAS (28), SHAM (28),	4 d	2 INC, CUT	-	X	X			**Acute**<br>-CAS: greater cortisol than SHAM at 60 min ($p < 0.001$)

Author (year)	Setting/ Groups (n)	Piglet age (n)	Technique(s) (n)	Other procedures performed (n)	Investigated parameters B&V	PP	WH	G&V	Main findings
	a. o.								**Short-term**<br>-No treatment differences were significant at 180 min (cortisol)<br>-CAS increased frequency of pain-related behavior in the first 30 min than SHAM: more isolated and inactive than SHAM<br>-No behavioral differences at 60 min
Hay *et al.* (2003)	CAS (36), SHAM (24), CON (24)	5 d	2 INC, PULL	-	X	X			**Short-term**<br>-CAS: reduced activity at udder and more inactive ($p < 0.001$) during the first 2.5 h compared to CON<br>-CAS: more pain-related behaviors for at least 2 d (e.g. tail wagging, rump scratching); reduced social cohesion ($p < 0.001$); desynchronization ($p < 0.05$).<br>-No clear effect on urinary corticosteroids and catecholamines<br>-Growth performance did not differ
Hofmann *et al.* (2019)	CAS (24), SHAM (24), a. o.	3–6 d	2 INC, CUT	BS, AA		X	X	X	**Short-term**<br>-30 min: CAS highest cortisol<br>**Long-term**<br>-Wound healing: median 7 (d 1), 6 (d 7), 4 (day 14)<br>-No effect on growth and losses
Kattesh *et al.* (1996)	CAS (24), CON (12)	7 d (12), 14 d (12)	2 INC	BS		X			**Short- and long-term**<br>-CAS on d 7 or 14 no effect on albumin, protein, cortisol in plasma<br>-Unbound cortisol decreased in all piglets over sampling period
Kielly *et al.* (1999)	CAS (241), CON (241)	3 d (248), 10 d (234)	2 INC	-				X	**Short-term**<br>-CAS at 3 d less weight gain on 1st d ($p = 0.01$) and 3rd d ($p = 0.06$)<br>-Weight gain not different between late CAS and CON<br>**Long-term**<br>-By weaning no differences between groups
Kluivers-Poodt *et al.* (2012)	CAS (32), SHAM (32), a. o.	2–5 d	1 INC, CUT	BS	X	X		X	**Acute**<br>-CAS: longer calls than SHAM<br>**Short-term**<br>-Litter effect on weight and growth, no treatment effect.<br>-no effects for glucose and creatine kinase

Author (year)	Setting/ Groups (n)	Piglet age (n)	Technique(s) (n)	Other procedures performed (n)	Investigated parameters				Main findings
					B&V	PP	WH	G&V	
Kluivers-Poodt *et al.* (2013)	CAS, SHAM, CON, a. o. (total: 144)	2–5 d	1 INC, CUT	-	X		X	X	**Short-term**<br>- Behavior changes with age, independent of treatment<br>- No treatment effect on growth; wound healing was rapid in all treatments<br>- CON: more ($p < 0.05$) No pain-related behavior than CAS
Langhoff *et al.* (2009)	CAS (38), SHAM (45), a. o.	4–6 d	2 INC, CUT	NaClA, BS	X	X			**Short-term**<br>- CAS: higher cortisol at 30 min, 1 h, 4 h<br>- CAS: more tail wagging and hanging tail
Lecchi *et al.* (2020)	CAS (12), SHAM (12), a. o.	4 d	2 INC, PULL	TD, IA, SS		X			**Short-term**<br>- CAS: cortisol increased after 30 min<br>- Pain related genes identified in CAS: abundance increase of miR-19b, miR-27b, miR-365; no differences in SHAM
Leidig *et al.* (2009)	CAS (18), SHAM (25), a. o.	3–4 d	2 INC, CUT	-	X				**Acute**<br>- Call duration enhanced from pre-treatment to CAS<br>- CAS: highest duration and intensity of defense behavior<br>- Not possible to separate effects of handling and castration
Lessard *et al.* (2002)	CAS (6), SHAM (6), a. o.	3 d, 10 d, 17 d	2 INC, CUT	BSAA		X		X	**Long-term**<br>- No effect on BW<br>- 3 d old: weaker antibody response to injected bovine serum albumin than in older groups<br>- No difference in antibody response in 3 d CAS and 3 d SHAM, but in 10 and 17 CAS weaker than 10 and 17 SHAM
Llamas Moya *et al.* (2008a)	CAS (20), SHAM (20)	5 d	2 INC, CUT	BS	X	X			**Short-term**<br>- CAS: pain-related behaviors ($p < 0.001$) up to 4 d, immediately after castration (e.g. huddling up, spasms, trembling); less walking ($p < 0.05$); more isolated and desynchronized ($p < 0.1$)<br>- No effect on plasma levels of TNFa, IL-1b, CRP, SAA and Hp<br>- CAS: higher cortisol than SHAM ($p < 0.1$)
Llamas Moya *et al.* (2008b)	CAS (32), CON (64)	5 d	2 INC, CUT	-	X	X		X	**Long-term**<br>- CAS reduced occurrence of sickness behaviors induced by low-dose endotoxin challenge<br>- No effect on inflammatory cytokines, acute phase proteins and cortisol

Author (year)	Setting/ Groups (n)	Piglet age (n)	Technique(s) (n)	Other procedures performed (n)	Investigated parameters				Main findings
					B&V	PP	WH	G&V	
Lomax *et al.* (2017)	CAS (10), SHAM (10), a. o.	3–5 d	2 INC, CUT	-	X				**Acute**<br>- Response scores not different immediately after CAS<br>**Short-term**<br>- Response increased significantly with weight of stimulation<br>- CAS: greater responses 2–4 h following castration ($p < 0.001$)<br>- Increase in response over 4 h in CAS ($p < 0.001$)
Lonardi *et al.* (2015)	CAS, SHAM	4 d	2 INC, CUT	IA	X	X			**Short-term**<br>- Higher cortisol in CAS after 20 min<br>- higher eye and rectal temperature in CAS after 3h<br>- alterations in posture and walking in CAS
Marchant-Forde *et al.* (2009)	CAS (32), SHAM + blood (16), SHAM (16)	2–3 d	CUT (16), PULL (16)	BS	X	X	X	X	**Acute**<br>- PULL longer duration than CUT ($p < 0.05$)<br>- CUT and PULL: more squeals, grunts, and escape attempts ($p < 0.05$) than in SHAM groups; no differences when time considered<br>**Short- and long-term**<br>- β-endorphin greater at 45 min in CUT<br>- Growth rates in PULL less ($p < 0.10$) than SHAM, CUT intermediate
Marchant-Forde *et al.* (2014)	CAS (40), SHAM + blood (20), SHAM (20)	2–3 d	CUT (20), PULL (20)	TR (CLIP or GRIND), IA (INJ or ORAL), ID (EN or ET), TD (HOT or COLD), BS	X	X	X	X	**Acute**<br>- CUT longer duration than PULL<br>- CAS more vocalization, escape attempts than SHAM ($p < 0.05$)<br>- PULL: fewer squeals per sec than SHAM + blood ($p < 0.05$)<br>**Short- and long-term**<br>- No differences in lesion scores between Less Stressful (CUT) and More Stressful (PULL)<br>- D 2–7: growth rate of More Stressful lower than SHAM ($p < 0.05$), Less Stressful intermediate<br>- Cortisol higher in CAS than SHAM
Marx *et al.* (2003)	CAS, SHAM, a. o. (total: 70)	7 d (28), 13 d (16), 19 d (26 d)	EM	-	X				**Acute**<br>- CAS: highest number of calls; higher screams<br>- Increased number of screams 30–110 s after start in CAS
McGlone *et al.* (1993)	CAS (455), CON (389)	1 d, 5 d, 10 d, 14 d, 15 d, 20 d	2 INC, PULL	EN, CLIP, IA (d 1)				X	**Short- and long-term**<br>- Exp. 1: No age effect on behavior:<br>- Reduced suckling, standing, more lying in CAS regardless of age<br>- Exp. 2: growth did not differ between CON and CAS

Author (year)	Setting/ Groups (n)	Piglet age (n)	Technique(s) (n)	Other procedures performed (n)	Investigated parameters				Main findings
					**B&V**	**PP**	**WH**	**G&V**	
									- CAS 14 d heavier at weaning than CAS 1 d
Morrison and Hemsworth (2020)	CAS, SHAM, a. o. (total: 288)	2 d	2 INC, CUT	BS	X	X			**Acute**<br>- Duration of vocalization and escape attempts greater during CAS than SHAM and TD<br>**Short-term**<br>- Higher cortisol after 15 min in TD and CAS<br>- 30 min: cortisol only higher in COLD and CAS<br>- More pain behavior in TD and CAS at 60 min, but no treatment effect after 24 h
Pérez-Pedraza *et al.* (2018)	CAS (24), SHAM (12), a. o.	5 d	1 INC (12), 2 INC (12), CUT	BS		X	X		**Acute**<br>- Increases in lactate and hematocrit immediately after CAS or SHAM<br>- CAS: reduced pH and HCO3, higher lactate and base excess alterations<br>- Metabolic acidosis greater in CAS (1 INC) than in CAS (2 INC)
Prunier *et al.* (2005)	CAS (6), SHAM (6), CON (6)	5–6 d	2 INC, CUT	BS		X			**Short-term**<br>- CAS: increases ($p < 0.05$) in adrenocorticotropin hormone, cortisol, and lactate (not in TD or TR)<br>- Adrenocorticotropin hormone higher in CAS than in SHAM and CON ($p < 0.05$), SHAM and CON similar ($p > 0.10$)<br>- Cortisol higher in CAS than in SHAM and CON, SHAM and CON similar ($p > 0.10$)<br>- Lactate: CAS > SHAM > CON ($p < 0.05$)
Puppe *et al.* (2005)	CAS (19)	14 d	EM	-	X				**Acute**<br>- Effect of CAS on call duration, peak frequency, pureness, entropy of sound)<br>- surgical period differed from postsurgical handling in all measures; presurgical handling differed only in pureness and entropy
Rauh *et al.* (2019)	CAS (24), SHAM (24), a. o.	3–6 d	2 INC, CUT	BS, AA		X	X	X	**Short-term**<br>- SHAM: fewer defensive movements during fixation ($p \leq 0.05$)<br>- During CAS: highest defensive movements at severing of cord<br>- Adrenalin and Noradrenalin rose in all groups, CAS: higher increase of Noradrenalin than SHAM ($p \leq 0.05$)

Author (year)	Setting/ Groups (n)	Piglet age (n)	Technique(s) (n)	Other procedures performed (n)	Investigated parameters				Main findings
					B&V	PP	WH	G&V	
Reynolds *et al.* (2020)	CAS (25), SHAM + IA (25), SHAM (25), a. o.	3–5 d	1 INC	IA	X				**Short-term**<br>-CAS: Longer navigation through chute after 25 min ($p < 0.001$)<br>-Higher cortisol in CAS after 1 h
Saller *et al.* (2020)	CAS (12), SHAM (11), a. o.	3–7 d	2 INC, CUT	NaClA	X	X			**Acute**<br>-Arterial blood pressure higher in CAS during incision and cutting of cords than SHAM ($p < 0.001$)<br>-CAS: higher heart rate after cutting of cords than SHAM ($p < 0.001$)<br>-Cortisol not different between groups, but within groups higher values after 20 and 40 min ($p < 0.001$)
Schön *et al.* (2006)	CAS (19)	14 d	EM	-	X				**Acute**<br>-During CAS: longer, higher, purer vocalization<br>-Lower peak frequency after surgery than before ($p < 0.001$).
Sutherland *et al.* (2010)	CAS (10), SHAM (10), a. o.	3 d	2 INC, PULL	BS	X	X		X	**Acute**<br>-Vocalizations greater ($p < 0.05$) in CAS compared to CON<br>**Short-term**<br>-Leukocyte counts not different among treatments<br>-Cortisol elevated ($p < 0.06$) in CAS compared to SHAM<br>-Bodyweight not different after 24 h
Sutherland *et al.* (2012)	CAS (10), SHAM (10), a. o.	3 d	2 INC, CUT	BS	X	X		X	**Acute**<br>-More stress vocalizations in CAS<br>**Short-term**<br>-CAS more time lying without contact at 30 min<br>-Higher cortisol in CAS after 30, 60, 120 min<br>-C reactive protein tended to be higher in CAS<br>-After 24 h no difference in body weight
Sutherland *et al.* (2017)	CAS (9), SHAM (9), a. o.	3 d	2 INC, CUT	BS	X	X			**Acute**<br>-More vocalizations in CAS than in SHAM ($p = 0.004$)<br>-Tail movement and behavioral scores greater ($p \leq 0.01$) in CAS than in CON<br>**Short-term**<br>-Cortisol elevated ($p < 0.05$) in CAS compared to CON<br>-CAS: more time lying without contact than CON; no effect on other behaviors

Author (year)	Setting/ Groups (n)	Piglet age (n)	Technique(s) (n)	Other procedures performed (n)	Investigated parameters				Main findings
					B&V	PP	WH	G&V	
Taylor and Weary (2000)	CAS, SHAM (total: 139)	7–13 d	2 INC, CUT, PULL	-	X				**Acute**<br>- CAS: incision more high-frequency calling than restraint in SHAM<br>- PULL/CUT of spermatic cords: greatest amount of calling, more than incision, regardless of order of events ($p < 0.001$)<br>- No difference in calling between PULL and CUT
Taylor *et al.* (2001)	CAS, SHAM (total: 84)	3 d, 10 d, 17 d	2 INC, CUT	-	X				**Acute**<br>- CAS: 3 times more high and low frequency calls than SHAM<br>- Rate of high frequency calling lower for youngest pigs; but no age by treatment interaction<br>**Short-term**<br>- 2 h after CAS: more sitting, standing, less lying<br>- During 22 h, CAS more time at udder, less lying down
Übel *et al.* (2015)	CAS, CAS + TD + ET, SHAM, a. o.	3–4 d	2 INC, CUT	TD (COLD), ET, IA, BS	X	X			**Short-term**<br>- CAS: higher cortisol after 30 and 60 min than SHAM ($p < 0{,}05$)<br>- Higher cortisol for 4 h after CAS + TD + ET than after CAS and SHAM<br>- Weights not different 4 d after SHAM, CAS and CAS + TD + ET
van Beirendonck *et al.* (2012)	CAS (total: 295)	1 d	1 INC, CUT	ET, TR, TD, IA	X		X	X	**Short-term**<br>- Differences in behavior, but weight at weaning not different<br>- Mortality higher in lightest piglets
Viscardi *et al.* (2017)	CAS + TD (2), CAS + TD + NaClA (5)	5 d	1 INC, PULL	TD (COLD), IA	X				**Short-term**<br>- More inactive behaviors up to 6 h post-CAS and TD<br>- Behavioral changes up to 7 h post-CAS compared to baseline<br>- Higher grimace scores at 0, 3, 4, 5 h post-CAS compared to scores at 7 h
Viscardi and Turner (2018a)	CAS + NaCl (15), SHAM (15), a. o.	5 d	2 INC, PULL	NaClA	X				**Short-term**<br>- More pain behaviors and tail wagging in CAS ($p < 0.05$)<br>- Increased pain behavior in CAS after 24 h
Viscardi and Turner (2018b)	CAS + NaCl (15), SHAM (15), a. o.	5 d	2 INC, PULL	NaClA	X				**Short-term**<br>- More tail wagging and pain behavior in CAS<br>- More pain behaviors after 24 h
Viscardi *et al.* (2020)	CAS (20), SHAM (10)	2 d	1 INC, CUT (10) or LASER (10)	-	X	X	X		**Short-term**<br>- Laser-CAS: more pain behaviors than scalpel-CAS ($p = 0.05$)<br>- Laser-CAS: more agonistic behavior than scalpel-CAS ($p = 0.005$) and SHAM ($p = 0.036$)

Author (year)	Setting/ Groups (n)	Piglet age (n)	Technique(s) (n)	Other procedures performed (n)	Investigated parameters				Main findings
					B&V	PP	WH	G&V	
									- No difference in wound healing or blood parameters
Weary *et al.* (1998)	CAS, SHAM (total: 102)	8–12 d	2 INC, CUT	-	X				**Acute**<br>- CAS: more high frequency calls than SHAM<br>- Exp. 1: greatest differences between CAS and SHAM during severing of spermatic cords<br>- Exp. 2/3: CAS high frequency calls at faster rates than SHAM
White *et al.* (1995)	CAS (86), a. o.	1 d, 2 d, 4 d, 8 d, 16 d, 24 d (26 ≤ n ≤ 30)	2 INC, CUT	-		X			**Short-term**<br>- CAS: higher heat rate, higher vocalizations<br>- Severing of spermatic cord: greater heart rate response<br>- Castration without anesthetic greater stress for pigs 8 d or older<br>- respiration rate: no variable measure of stress associated with castration
Yun *et al.* (2019)	CAS (29), SHAM (29), a. o.	5 d	2 INC	-	X				**Acute**<br>- Pain behavior during castration<br>- More tail wagging and aggressive behavior in SHAM<br>**Short-term**<br>- Behavioral changes after castration, abnormal behaviors<br>- CAS induced changes in behavioral measures at 0 h<br>- Inactive standing or sitting, tail wagging and aggressive behavior differed between CAS and SHAM at 0 h
Zöls *et al.* (2006)	CAS (48) SHAM (46), a. o.	4–6 d	2 INC, CUT	BS		X			**Short-term**<br>- CAS: higher cortisol after 1 and 4 h ($p \leq 0.05$) than SHAM<br>- No difference in cortisol after 28 h

AA = analgesia administration; a. o. = and others; BS = blood sampling; BSAA = bovine serum albumin application; B&V = Behavior & vocalization; CAS = castration; CON = control piglets (not handled); d = days; EM = emasculator; EN = ear notching; ET = ear tagging; G&V = growth & vitality; HRV = heart rate variability; IA = iron application; INC = incision (1 = horizontal; 2 = vertical); INJ = injection; m = months; NaClA = NaCl application; PP = physiological parameters; SHAM = sham-handled piglets; SS = saliva sampling; TD = tail docking; TR = teeth resection; w = weeks; WAO = water administration orally; WH = wound healing.
